# Psiscan: a computational approach to identify H/ACA-like and AGA-like non-coding RNA in trypanosomatid genomes

**DOI:** 10.1186/1471-2105-9-471

**Published:** 2008-11-05

**Authors:** Inna Myslyuk, Tirza Doniger, Yair Horesh, Avraham Hury, Ran Hoffer, Yaara Ziporen, Shulamit Michaeli, Ron Unger

**Affiliations:** 1 Faculty of Life Science, Bar-Ilan University, Ramat-Gan, 52900, Israel; 2 The Department of Physics of Complex Systems, The Weizmann Institute of Science, Rehovot, 76100, Israel

## Abstract

**Background:**

Detection of non coding RNA (ncRNA) molecules is a major bioinformatics challenge. This challenge is particularly difficult when attempting to detect H/ACA molecules which are involved in converting uridine to pseudouridine on rRNA in trypanosomes, because these organisms have unique H/ACA molecules (termed H/ACA-like) that lack several of the features that characterize H/ACA molecules in most other organisms.

**Results:**

We present here a computational tool called Psiscan, which was designed to detect H/ACA-like molecules in trypanosomes. We started by analyzing known H/ACA-like molecules and characterized their crucial elements both computationally and experimentally.

Next, we set up constraints based on this analysis and additional phylogenic and functional data to rapidly scan three trypanosome genomes (*T. brucei*, *T. cruzi *and *L. major*) for sequences that observe these constraints and are conserved among the species. In the next step, we used minimal energy calculation to select the molecules that are predicted to fold into a lowest energy structure that is consistent with the constraints. In the final computational step, we used a Support Vector Machine that was trained on known H/ACA-like molecules as positive examples and on negative examples of molecules that were identified by the computational analyses but were shown experimentally not to be H/ACA-like molecules. The leading candidate molecules predicted by the SVM model were then subjected to experimental validation.

**Conclusion:**

The experimental validation showed 11 molecules to be expressed (4 out of 25 in the intermediate stage and 7 out of 19 in the final validation after the machine learning stage). Five of these 11 molecules were further shown to be bona fide H/ACA-like molecules. As snoRNA in trypanosomes are organized in clusters, the new H/ACA-like molecules could be used as starting points to manually search for additional molecules in their neighbourhood. All together this study increased our repertoire by fourteen H/ACA-like and six C/D snoRNAs molecules from *T. brucei *and *L. Major*. In addition the experimental analysis revealed that six ncRNA molecules that are expressed are not downregulated in CBF5 silenced cells, suggesting that they have structural features of H/ACA-like molecules but do not have their standard function. We termed this novel class of molecules AGA-like, and we are exploring their function.

This study demonstrates the power of tight collaboration between computational and experimental approaches in a combined effort to reveal the repertoire of ncRNA molecles.

## Background

Non-coding RNAs (ncRNA) are RNA molecules that range in size from less than a hundred to thousands of nucleotides. These RNAs are transcribed but are not translated. One of the largest classes of ncRNA in eukaryotes are the snoRNA (small nucleolar RNA) named based on their localization to the nucleolus. Such molecules also exist in Archaea, in which they are termed sRNA [1,2].

The snoRNA class is divided into two major families, C/D and H/ACA. Their names specify the conserved boxes they carry. SnoRNAs guide modifications on other RNA molecules, but also function in rRNA processing. C/D snoRNAs serve as guide RNAs for 2'-*O*-methylation and H/ACA for isomerization of uracil to pseudouridine [3-5]. Both the C/D and H/ACA snoRNAs bind to the site of modification by direct base pairing with the target RNA. The majority of these guide RNAs are responsible for the modification of ribosomal RNA (rRNA), and in some cases of small nuclear RNAs (snRNAs) [6-10]. Other modification targets include transfer RNAs in Archaea [1], spliced leader RNAs in trypanosomes [11], and at least one brain-specific mRNA in mammals, serotonin pre-mRNA, in which snoRNA regulate alternative splicing [12]. Spliceosome function also depends on the modification of snRNAs by C/D and H/ACA RNAs, since these modifications exist in the U snRNA domains that are involved in the RNA-RNA interactions that take place during the splicing reaction [13]. In addition, another special H/ACA RNA in mammals is telomerase RNA, which is required for telomere synthesis [14].

Most relevant to this study are H/ACA snoRNAs. H/ACA molecules in most eukaryotes consist of two hairpins, a 5' hairpin followed by a single-stranded domain called the H-box (with the sequence ANANNA, where N stands for any nucleotide), and a 3' hairpin followed by an ACA-box. The snoRNA's pseudouridylation pocket consists of two short sequences that are complementary to the residues that flank the target uridine to be converted to pseudouridine. The uridine residue, which undergoes pseudouridylation, is located 14–16 bp upstream from the H or ACA box. The 5' hairpin and the 3' hairpin are similar in their structure and function. These H/ACA have the potential to guide modification of two different rRNA targets [5,7]. Even when the RNA only contains a single guide sequence, the two hairpin domains are essential for activity [15]. Four core proteins, namely, Gar1p, Nop10p, Nhp2p, and Cbf5p/dyskerin, were identified to form the eukaryotic H/ACA snoRNP (RNA-Protein complex) [16-22]. The crystal structure of a functional complex of these four highly conserved proteins in complex with a single-hairpin H/ACA snoRNA was solved in *Archaea *[23].

Trypanosomes are unicellular parasitic protozoa that are the causative agent of several infamous parasitic diseases including African trypanosomiasis caused by *Trypanosoma brucei*, Chagas' disease caused by *Trypanosoma cruzi*, and Leishmaniasis caused by leishmania species. Trypanosomatids diverged early in the evolution of eukaryotes [24] and are well-known for harboring exotic and unique RNA processing mechanisms such as nuclear pre-mRNA trans-splicing [25] and mitochondrial RNA editing [26]. In addition, the large rRNA subunit is synthesized as a single RNA molecule that then undergoes specific cleavages that yield two large rRNA molecules and four small RNAs, ranging in size from 76 to 220 nt [27].

In trypanosomes, only single-hairpin H/ACA molecules which lack the H-box and have an AGA-box instead of ACA-box were described [28]. Following our previous notation, throughout this paper we refer to these molecules, i.e. single hairpin molecules with AGA-box and a regular H/ACA function as determined by their destabilization in CBF5 knockdown cells (see below) as **H/ACA-like**. The novel class of molecules that share the same structural features but are not destabilized in CBF5 knockdown cells are referred to as **AGA-like **(see below).

Since the discovery of single-hairpin H/ACA-like molecules in trypansomes, similar single hairpin RNAs have been discovered in Archea [20,29], and Euglena [30]. Single-hairpin H/ACA-like molecules in trypanosomes operate in a similar fashion as their double-hairpin counterparts.

The organization of trypanosome snoRNAs most closely resembles that of plants [31], as their coding genes are clustered, and each cluster carries a mixture of both C/D and H/ACA-like RNAs [32,33]. The trypanosome snoRNAs are processed from long polycistronic transcripts [34,35], but the machinery that executes this processing is currently unknown. The clusters are usually repeated several times in the genome [28,35-37], and could appear at a second location within the same chromosome [32].

Prior to the current study, 60 C/D snoRNA and 34 H/ACA-like snoRNA molecules, which have the potential to direct 87 methylations and 32 pseudouridylations, respectively, were identified in *Trypanosoma brucei *[33,38] and 62 C/D and 37 H/ACA-like snoRNAs that can potentially guide 79 methylations and 30 pseudouridylations, respectively, were identified in *Leishmania major *[32].

Early studies suggested the existence of at least 100 2'-*O*-methylated nucleotides on the rRNA of the trypanosomatid, *Crithidia fasciculata *[39], and recent results in *Euglena*, which is closely related to trypanosomes, suggest the presence of at least 200 Nms on its rRNA[40]. The latest mapping data of methylation sites in *T. brucei *suggest that there are at least 131 Nms [38]; therefore the C/D snoRNAs identified so far in *T. brucei *and *L. major *(which can guide two modification each) are sufficient to guide about 70% of the expected modifications.

While the complete set of 24 H/ACA-like snoRNAs that guide 44 pseudouridylations in yeast was identified [41], the number of pseudouridines in the rRNA of trypanosomatids is still unknown. Based on the fact that the number of C/D molecules in the known clusters is double that of the number of H/ACA-like molecules, and clusters consisting only of H/ACA-like molecules were not identified to date, it is expected that about 70–80 pseudouridine sites should exist on the rRNA of these parasites. Thus, we estimate that only about 40% of the expected repertoire of pseudouridylation sites has been identified to date.

Laboratory techniques for the genome wide identification of RNA molecules are expensive, time-consuming, and labor-intensive. In addition, such experimental methods have a bias toward highly abundant molecules. Thus, the study of ncRNA molecules has advanced thanks to a combination of *in silico *and experimental methods, usually through an approach in which predictions made by computational algorithms such as those described in [2,42-48] are then validated by experimental work.

For C/D guide snoRNAs, the presence of relatively well conserved box motifs and 10–21 nt complementary between the guide RNA and its target has enabled the development of successful computational screens, such as the SnoScan program [44]. In contrast to C/D guide snoRNAs, the H/ACA guide snoRNAs have only two primary sequence motifs, the "H-box" and the "ACA-box", the H/ACA hairpin secondary structures exhibit considerable variation, and the target-guide duplexes are of varying lengths and may be imperfectly paired, making the design of an effective computational screen for H/ACA snoRNAs and their associated pseudouridylation sites significantly more difficult. Still, several computational predictors were shown to yield significant results in identifying candidate H/ACA snoRNA sequences in eukaryotic organisms in which H/ACA molecules fit the consensus H/ACA features, including a two hairpin structure [45,49-53].

Some of these studies need to be mentioned as they are relevant to our work: SnoGPS [51] was designed to search for the classic double hairpin H/ACA molecules in eukaryotic genomic sequences, but can be configured to run in single-stem mode as well. We will show in the Discussion session that SnoGPS can not be used, as a stand alone program, to search for H/ACA-like molecules in trypanosomes.

Muller et al. [54,55] have devised a specific algorithm to search for H/ACA in Archaea which are single stem. In Pyrococcus genomes this algorithm was shown to be effective in detecting the small repertoire of H/ACA molecules in these organisms. However the method is based on constraints which are specific to Archaeal H/ACA (like the presence of K-Turns and K-Loops and the existence of many G:C pairs in stems), and are not present in trypanosomes.

In a very recent paper Hertel et al. [53] used a machine learning approach (SVM – Support Vector Machine) to detect snoRNA molecules whose ribosomal target is unknown. While the approach was very successful in identifying known H/ACA in several organisms (Human, Nematodes and Drosophila) it failed to detect any of the known H/ACA-like molecules in the trypanosomatid genome of *L. major*.

Thus, it is clear that computational detection of H/ACA molecules is more difficult for the single-hairpin molecules than for the standard double-hairpin form. The double-hairpin structure with its characteristic spacer length between the stems is a dominant feature that may be exploited by the computational algorithms. Since trypanosome H/ACA-like RNAs are composed of a single-hairpin and lack most of the features that have Archaeal H/ACA, we can not use neither the existing algorithms to search for H/ACA molecules from other eukaryotes nor programs that exploit the specific features of Archeal H/ACA. Therefore, we undertook the challenge of devising a novel algorithm to identify H/ACA molecules in these species.

The Psiscan algorithm described here is based on a pipeline that combines several computational approaches: Initial filtering based on trypanosome specific constraints followed by comparative analysis of trypanosome species followed by folding energy requirements and concluded in a SVM analysis.

Thus, it presents a systematic yet specific approach to detect novel single-hairpin H/ACA and AGA like molecules in trypanosome species. The algorithm was able to significantly increase the repertoire of these molecules in *T. brucei*.

All together the experimental validation revealed five novel H/ACA-like as well as six novel non-coding RNA species that possess many H/ACA-like features; the latter group were not classified as H/ACA-like because their level did not change in *T. brucei *cells silenced for the pseudouridine synthase, CBF5 [56]. In addition, bioinformatic analysis of the genomic loci of the five novel H/ACA-like ncRNAs led to the identification of seven additional novel H/ACA-like and six novel C/D snoRNA molecules. We also identified two orthologous clusters in *L. major *adding 2 H/ACA and 2 C/D molecules to the *L. Major *repertoire.

Thus, our study suggests that trypanosomes carry numerous ncRNA molecules of yet unknown function. We suggest that the novel molecules discovered in this study that share structural features with H/ACA-like molecules but are not affected by silencing, might be similar to "chimeric" molecules like scaRNA [10] and telomerase RNA [14] which are known to have H/ACA domains. These molecules have been found in vertebrates but not yet in trypanosomes. Experiments are underway to characterize these novel molecules.

## Methods

### Extraction of *T. brucei*, *T. cruzi *and *L. major *genomes

The *Trypanosoma brucei *genome release 4 [57], *Leishmania major *genome release 5 [58] and *Trypanosoma cruzi *genome release 4 [59] were downloaded from GeneDB  and used for analysis and prediction of H/ACA-like snoRNA.

### Collection of H/ACA-like snoRNA repertoire

The repertoire of 34 H/ACA-like snoRNA sequences and their rRNA target sequences was taken from Liang et al. (2005) in *T. brucei*. Similar data for 37 H/ACA-like sequences in *L. major *were taken from Liang et al. (2007). Finally, 29 H/ACA-like snoRNAs (unpublished data) were found in the *T. cruzi *genome by homology search using Blast [60].

### Secondary structure prediction by MFOLD

Secondary structure prediction and minimum free energy (ΔG) calculation of the predicted structure were performed by the MFOLD program (version 2.3) [61].

### Bioinformatic analysis using Support Vector Machine

Support Vector Machine (SVM) is a machine learning technique that is used for classification and regression. Recently, it was shown to be useful in bioinformatic studies [62]. We applied the Support Vector Machine algorithm [63] using the SVMlight implementation in classification mode [64]. The SVM was used with a linear basis function to rank the candidate H/ACA-like snoRNAs that were predicted in the first stages of the analysis. For each candidate sequence the decision function gives a score which can range between -8 and 8 according to the similarity of a candidate to the negative or positive training group. The higher the score is (from 0 to +8), the more similar the features of the test sequence to the features of the real H/ACA snoRNAs. Consequently, sequences that have a low score (from 0 to -8) are more similar to the false H/ACA candidates. More details on the specific way in which the SVM method was used are given in the Results.

### Ten fold cross validation and evaluation of the prediction accuracy

The validation process was run one hundred times; each time the positive and negative training data were randomly grouped into ten subsets of approximately equal size. Then, nine subsets of the positive group and nine subsets of the negative group were used as training data and the remaining two subsets constitute the test set. For each run, the SVM was trained on the randomly selected training set and then performed the classification process on the test set. As usual we define positive samples in the test set classified as positive as True Positives, and their number is denoted as TP. Negative samples classified to be negative are called True Negative and their number is counted by TN. Negative samples that were wrongly classified as positives are False Positives and counted by FP. Positive samples that were wrongly classified as negatives are considered False Negatives and are counted by FN. To combine these four categories into one measure we used the standard notation of accuracy which is defined by accuracy = $\frac{TP+TN}{TP+TN+FP+FN}$. We report the average accuracy for the hundred validation processes.

### Enrichment factor (EF)

While the goal of this work is to detect new unknown H/ACA-like molecules in trypanosomes, we can use the set of known H/ACA-like molecules in trypanosome as a benchmark to trace the performance of our computational pipeline. I.e. in each stage of the pipeline we can check how many of the known H/ACA-like molecules are still included in the candidate list produced by the algorithms. Towards this end we defined the Enrichment Factor (EF) as the ratio between the baseline frequency of known H/ACA molecules in the genome to their frequency in the various candidate sets. There are 34 known H/ACA-like molecules in the *T. brucei *genome. As AGA is a mandatory box for these molecules we calculated how many distinct sequences of length 70 (which is the average length of these molecules) appear in the genome that end with AGA. As such sequences can overlap, we defined a sequence to be distinct if its overlap with another sequence is not more than 20 nts. Using a simple greedy counting we found that the *T. brucei *genome contain 450,965 such molecules. Hence the baseline frequency of H/ACA-like molecules in T. Brucei is 34/207339 = 0.000075.

### Construction of h2 mutations using PCR mutagenesis

Tagging of h2 H/ACA was performed by PCR mutagenesis using primers carrying the mutatant tag sequence. The primers used to introduce the mutations and the primers used for the amplification of the mutant fragments for cloning into the pX expression vector are specified in the Additional File 1. The mutations were confirmed by DNA sequencing. The mutations were cloned into the pX-neo episomal vector, which carries a neomycin resistance gene (neo). Stable cell lines carrying the different constructs were selected on 25 μg/ml neomycin and lines expressing high levels of the tagged molecule were selected on 500 μg/ml neomycin. The stable cell lines carrying the different constructs were established as previously described [65].

### Primer extension analysis

Primer extension was performed using end-labeled oligonucleotides (10^5 ^cpm/pmol) [see Additional file 2]. After annealing at 60°C for 15 min, the sample was kept on ice for 1 min. 1 unit of reverse transcriptase (Expand, RT, Roche Molecular Biochemicals) and 1 unit of RNase inhibitor (Promega) were added, and extension was performed at 42°C for 90 min. The reaction was analyzed on a 6% polyacrylamide denaturing gel next to end labeled pBR322 HpaII digest.

## Results

### Manual analysis of 100 known H/ACA-like and construction of a heuristic model

**Figure 1:**
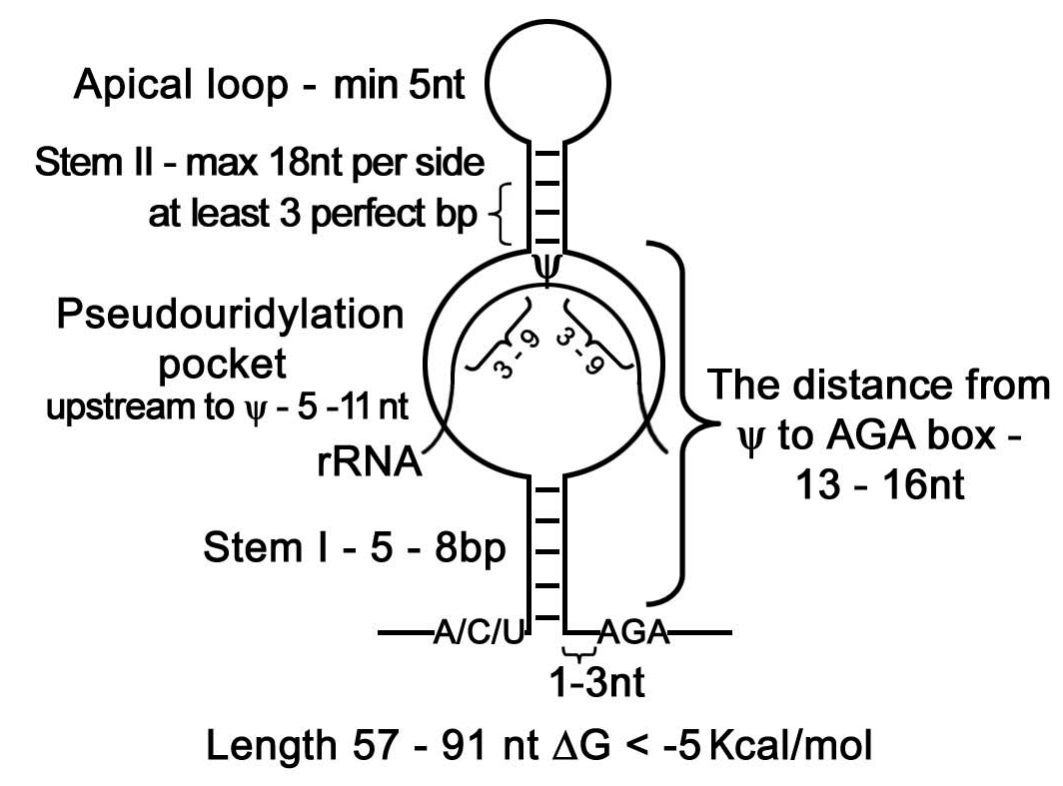
**The consensus structure of trypanosomatid H/ACA-like snoRNA**. The single hairpin structure consists of stem I, a loop that contains the pseudouridylation pocket, stem II, and an apical loop. The target rRNA is bound within the pseudouridylation pocket. The AGA box is found immediately downstream of the hairpin. The modified uridine on rRNA is marked by Ψ.

As an initial step in designing our computational identification protocol, we compiled all the 100 known H/ACA-like snoRNAs from *T. brucei*, *L. major *and *T. cruzi*. These sequences were folded by MFOLD with constraints that fit the general description of single stem-hairpin structure composed of two stems and two loops (the pseudouridylation pocket and the upper loop). We enforced features to forbid base-pairing with the AGA box and with the sequences that constitute the pseudouridylation pocket. In addition, we required that two nucleotides from stem II immediately following the pseudouridylation pocket be base paired (See Fig 1).

In all trypanosomatid H/ACA-like snoRNAs known so far, the terminal box sequence is AGA, not ACA, and is located 3 nt upstream from the 3' end. In addition, all of the H/ACA-like RNAs can form only a single stem-loop structure with sizes ranging from 57 to 91 nt [32,33]. The 5' ends of H/ACA-like snoRNAs are usually situated 1 to 3 nt upstream from the stem and in most cases, an A exists 1 nt upstream of stem I (91% in *T. brucei*, 86% in *T. cruzi *and 66% in *L. major*). C can also appear in this position (about 30% of the time in *L. major*, 14% in *T. cruzi *and only 6% in *T. brucei*), while U is rarely found and G was not observed in this position. Stem I has usually perfect base-pairing and ranges from 5 to 8 nt in length. Usually, one to three unpaired nucleotides exist between stem I and the AGA box. The pseudouridylation pocket varies in size: the upstream side to the pseudouridine varies in size from 5 to 11 nt, whereas the downstream side of the pocket depends on the size of the stem I and space between the stem I to AGA motif. Altogether, the size of downstream side to the modified uridine with stem I and unpaired nucleotides before the AGA box range in size from 13–16 nt, because the pseudouridine is always located 13–16 nt upstream from the AGA-box of the snoRNA as in all canonical H/ACA [4]. The target-guide duplexes of pseudouridylation pocket with rRNA target are of varying sizes from 3 to 9 nt on each side. Stem II also varies in size, but a perfect stem of 3 to 7 nt must exist immediately adjacent to the pseudouridylation pocket. The conserved sizes of the stems is supported by the presence of compensatory changes [32] between orthologs from *T. brucei*, *T. cruzi *and *L. major*. The apical loop is less conserved among the orthologs, but has a minimum size of 5 nt. The predicted secondary structures suggest stable structures with minimal free energy (ΔG) of not higher than -5 Kcal/mol. Based on these features, a consensus structure for trypanosomatid H/ACA-like snoRNA was chosen (See Fig 1).

### Determining structural elements essential for stable expression of H/ACA-like RNA

**Figure 2:**
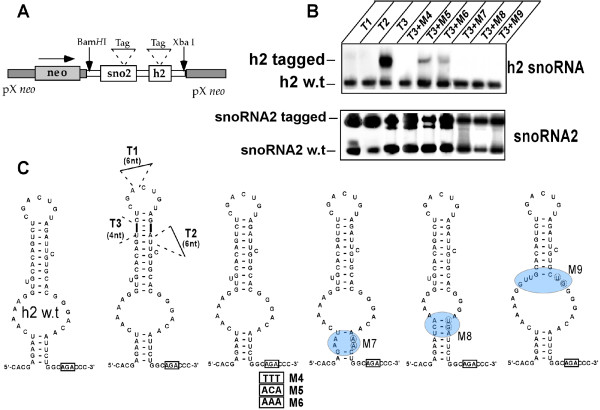
**Expression of mutants introduced into the H/ACA-like RNA h2 of *L. collosoma***. (A). Schematic presentation of the h2 construct. The two RNAs, sno2 and h2, were tagged to enable their expression to be examined on the background of wild-type RNA. The cluster was cloned into the pX expression vector (marked in gray) using *BamH*I and *Xba*I sites. (B). Primer extension analysis. RNA was extracted from transgenic lines carrying the different mutations shown in panel C. The RNA was extended with h2 and snoRN-2 specific oligonulceotides listed in the Additional File 1. The different cell lines carried a single mutation (T1 to T3) or the double mutations (T3 + one the M mutations are indicated). The position of the extension products for snoRNA-2 or h2 are indicated. (C). Schematic representation of the mutations introduced in h2 depicted on the secondary structure of the RNA. The positions on the introduced T mutation for tagging the molecules and the size of the insertion (in nt) are given. Mutations M4 to M6 indicate the sequence changes introduced into the ACA. The position of the mutations in M7 to M9 is highlighted with a circle and the exact nts that were changed are individually circled.

To identify the structural features that are essential for stable expression of H/ACA-like molecules, we used the *Leptomonas collosoma *system. We have previously identified several H/ACA-like RNA species in the snoRNA-2 locus in *L. collosoma *[66]. Expression of a tagged C/D RNA snoRNA-2 from this locus was established by cloning the gene with its flanking sequences into the pX expression vector [35]. For these experiments, we used a plasmid carrying the tag in the C/D snoRNA (sno2) that is carried on the same plasmid as h2. The expression of tagged sno-2 controls for expression, from the plasmid (copy number) and serves as a positive control for the expression of h2 mutants (See Fig 2-A). Mutations were introduced into the h2 RNA by PCR mutagenesis as described in Materials and Methods. After verifying the mutations, transgenic parasites expressing these mutations were selected by growth on elevated G418 concentration. We previously demonstrated that both the chromosomal snoRNA-2 as well as the tagged molecule are efficiently expressed in cell lines expressing the snoRNA [35]. The first set of mutations was introduced to tag the h2 molecule by inserting a tag of several nucleotides in either the apical loop or the upper part of stem 2. In addition, we inserted symmetrical complementary sequences in each side of the second stem, thereby lengthening the molecule. A schematic representation of the positions of these alterations is given in Fig. 2-C, and the expression of these tagged RNAs is shown in Fig 2-B. The expression was examined by primer extension using anti-sense oilgonucleotides complementary to the 3' end of the molecule that extends both the wild-type and the tagged RNA. The results suggest that h2 molecules can not tolerate insertion of nucleotides in either the apical loop or the second stem. Expression was only detected if a symmetrical complementary sequence was inserted in stem II. This tagged molecule was further used to introduce additional mutations and examine the importance of additional structural features of h2.

We next mutated the AGA sequence to either ACA or AAA (the mutations are depicted in Fig 2-C) and the expression by primer extension is presented in Fig 2-B. The results suggest that this AGA sequence can not tolerate changes, as such changes dramatically decrease expression. Note that the level of tagged snoRNA-2 serves as a positive control to demonstrate the high level of expression form the episomal pX plasmid. To further explore other structural features that may be important for the stable expression of these RNA species, three additional changes were introduced to the tagged h2 RNA molecule, as depicted in Fig 2-C. M7 stem I was shortened to two nucleotides by a symmetrical deletion, M8 stem I was extended to 9 nts, and finally the pesudouridylation pocket in M9 was destroyed by enlarging the loop by 6 nts. The results in Fig. 2-B suggest that none of these changes can be tolerated and that the tagged mutated RNAs were not stably expressed. These results suggest that: (1) The size of the apical loop is important for RNA stabilization; (2) Stem II can tolerate only symmetrical changes in its length; (3) Stem I must be longer than 2 nucleotides and not larger than eight nucleotides; (4) The pseudouridylation pocket can not tolerate extensions, even if these preserve the loop structure. These conclusions assisted us in choosing the constraints to be used by the algorithm to search for additional molecules in the genome.

### Psiscan: Genome wide search pipeline

**Figure 3:**
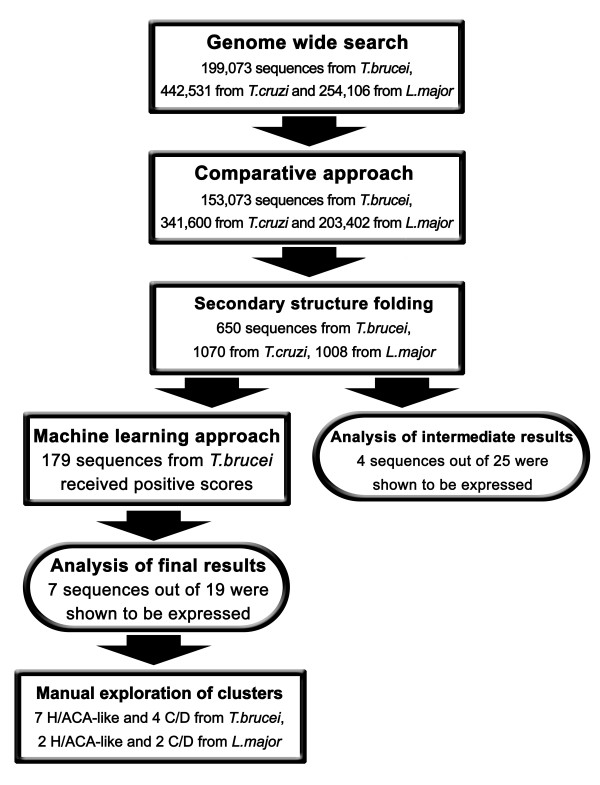
**Schematic representation of the genome wide search pipeline**. The computational stages are shown as rectangles and the experimental verification steps are presented as oval shapes.

Using the consensus description presented in Fig 1 and described above, we developed a search pipeline consisting of: (1) A genome wide search for sequences that carry H/ACA-like characteristics. (2) Homology prediction approach. (3) Folding into an appropriate secondary structure. The sequences that passed these three filters were further refined by (4) a machine learning approach and followed by (5) manual exploration of clusters. These five stages are presented schematically in Fig 3 and are detailed below:

### Genome wide search for sequences that fit the description

The first filter in the pipeline was a genome wide search, which scans the entire genome and detects sequences that fit the consensus model presented in Fig 1. The program receives as input the genome sequence, rRNA sequence and the parameters of the model, e.g. the range of H/ACA-like sizes, the size of stem I, the extent of base pairing with rRNA. The filter moves through the input genome and identifies each AGA sequence. Next, it tries to match stem I 5–8 nt upstream from the AGA with another 5–8 nt sequence at a distance of 49–83 nt upstream that can complement the first sequence. This was done by trying all possible ways to create a perfect stem I in length of 8–5 bp in distance of 83–49 nt upstream to the AGA. Each possible structure for stem I was passed to the next stage as a separate candidate to form H/ACA-like molecule. Note that G-U base pairing, which is common in RNA structures, is allowed.

For each sequence which has an AGA motif and a perfect stem I, the program tries to find the target rRNA by base pairing of the 3–9 nts that flank stem I on each side to the 6–18 nt on the rRNA again by trying all possible combinations. After matching the target to the sequence we check that the pseudouridylation pocket is of the right size, i.e. the length of the pseudouridylation pocket upstream to the Ψ varies in size from 5–11 nts and the length of the pseudouridylation pocket downstream to the Ψ is counted together with the size of stem I and number of unpaired nucleotides before the AGA box to be between 13–16 nt (see Figure 1). Then, the program tries to base pair perfectly at least 3–7 bp immediately after the pseudouridylation pocket and to extend this stem to the maximum of 18 nt in each side by non-perfect base pairing. Finally, it checks that there are no less than 5 nts remaining, which constitute the apical loop.

1,458,629 candidates from *T. brucei*, 3,349,031 candidates from *T. cruzi *and 1,847,073 candidates from *L. major *fit this crude model. Because of the way the genome search works, it is clear that the same sequence (or a sequence with a minor shift) may fit the model in many different ways. In order to check the real number of the different candidates, we checked the number of sequences by their genome location. Two sequences are considered different if they overlap by at most 20 nts. Using this measure we got 199,073 unique sequences from *T. brucei*, 442,531 from *T. cruzi *and 254,106 from *L. major*. 32 known H/ACA-like molecules from *T. brucei *were found in this set. Thus, their frequency is 0.00016 (32/199073), i.e. an Enrichment Factor of 2.1 compared to the baseline.

### Comparative approach

In order to filter the very large number of candidates, we required that each molecule have a homologue in at least one other trypanosome genome (out of the three genomes that we analyzed). Previous studies showed that the level of conservation in the upper stem and apical loop is not high [32], so in fact only about the first 20 nt of each molecule were considered relevant to detect similarity between the two RNAs. Two sequences were considered to be similar if they matched in at least 10 nt out of the 16 nt starting from the stem I of the 5' end of both sequences

In order to direct the algorithm to identify short conserved sequences which are not a part of a longer sequence, such as ribosomal RNA or proteins, we required that the sequences on the 10 nts flanking each side of the molecules (upstream to stem I of the 5' end and downstream immediately after AGA box) to be not conserved, thus a maximum of 4 matched nucleotides out of the stretch of 10 nts on each side was allowed.

663,561 candidates (merged to 153,073 unique sequences, see above) from *T. brucei*, 1,608,047 candidates (341,600 sequences) from *T. cruzi *and 922,894 candidates (203,402 sequences) from *L. major *were passed to the third filter in the pipeline.

31 known H/ACA-like molecules from *T. brucei *were found in this set. Thus, their frequency is 31/153073 = 0.0002 suggesting an Enrichment factor of 2.65 compared to the baseline.

### Secondary structure folding

We assume that true H/ACA-like molecules will have stable secondary structure and thus should have low free energy score predicted by RNA folding programs like MFOLD [61]. However, calculating *ab initio *the secondary structure of these molecules is both not reliable (prediction of the secondary structure of such short molecules is known to be problematic [67]) and highly time consuming. We therefore used the option of the MFOLD program that forces the predicted structure to follow user-defined constraints. Our constraints prevent base pairing between the AGA box and sequences which recognize the rRNA in the pseudouridylation pocket. We also forced two nucleotides from stem II immediately after the pseudouridylation pocket to be base paired [see Additional File 3].

The predicted secondary structure for certain sequences was considered satisfactory if it obeyed the consensus H/ACA-like secondary structure as described in Figure 1 and was energetically stable, defined as ΔG lower than -5 Kcal/mol. MFOLD computes all possible secondary structures for a certain sequence and returns as output the best fifty variant structures. Only when both sequences in each comparative pair had at least one satisfactory secondary structure, they were passed to the next stage of the pipeline. Note that as each sequence can be folded in more than one legitimate structure according to the requirements, there are more candidate structures than sequences.

At this stage, we were left with 3452 candidates (2189 sequences) from *T. brucei*, 10,369 candidates (6329 sequences) from *T. cruzi *and 6263 candidates (4137 sequences) from *L. major*. 15 known H/ACA-like snoRNAs from *T. brucei *were found. The Enrichment factor of this stage is 57.6 compared to the baseline.

Since sequences that are annotated as protein coding are less likely to harbor ncRNA molecules, we eliminated all sequences that came from such regions of their respective genome. We preferred to include the entire genome in the original search since we have previously detected C/D snoRNA in genomic loci annotated as hypothetical conserved proteins. Thus, in future studies, we may continue searching the entire list of candidates for additional H/ACA-like molecules that were omitted here. However, in the current study, we filtered out coding regions including hypothetical proteins at this stage and were left with 650 sequences from *T. brucei*, 1070 from *T. cruzi *and 1008 from *L. major*.

### Intermediate results filtering and analysis

**Figure 4:**
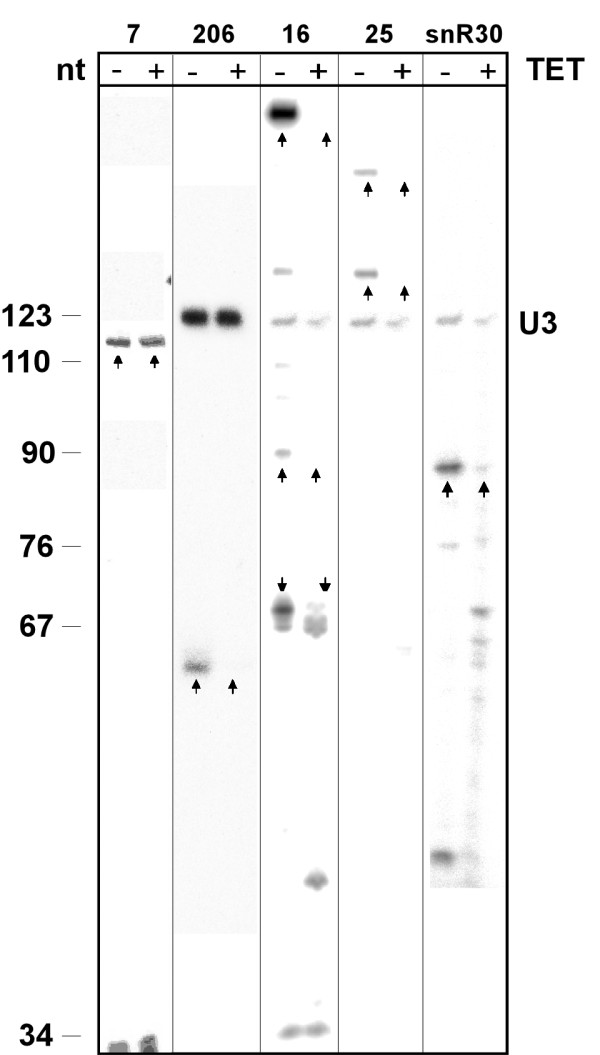
**Expression of putative H/ACA-like RNA**. RNA was subjected to primer extension with the oligonucleotides listed in the Additional File 2. Total RNA was extracted from cells before induction of the silencing of CBF5 (-Tet) or after 2 days of silencing (+Tet), as previously described (Barth et al., 2005). The extension products were separated on a 6% polyacrylamide 7 M urea gel next to labeled marker (pBR322 MspI digest). The size of the marker in nt is indicated. The lanes for the different candidates are marked by their identification number. To control for equal loading of samples, the RNA was extended with oligonucleotide complementary to U3 snoRNA. The relevant extension products are marked with arrows.

Since the validation of so many candidates in the laboratory is a labor intensive and costly process, we ranked the results from the *T. brucei *genome according to their level of conservation to both *L. major *and *T. cruzi*. The 25 most conserved sequences were tested for expression by primer extension analysis [see Additional File 4 for the list of the sequences]. Four sequences out of these 25 were shown to be expressed (Fig 4). However, two of the RNAs (7 and 25 on Fig 4) were larger than the expected size. In order to confirm that the four expressed RNAs are H/ACA-like, the level of the RNA was examined in CBF5 RNAi silenced cells before and after 3 days of silencing. We previously demonstrated that H/ACA-like RNA are destabilized in these silenced cells [56]. Three sequences (16, 206 and 25 on Fig 4) were sensitive to silencing, suggesting their role as H/ACA-like snoRNA; the function of the fourth one (number 7 on Fig 4) is currently unknown.

### Machine learning approach

In order to increase the sensitivity and specificity of the search and to rank the candidates according to the probability that they are indeed bona fide ncRNA molecules, we used a Support Vector Machine (SVM) [63].

The SVM receives, as input, vectors of features of the training data, selects a set of significant features to be used in the classification process, creates a model which consists of a combination of the significant features and then classifies the feature vectors of the test data according to the derived model. The training set for this program must include two different groups of the data – positive and negative. SVM creates a maximal separating hyperplane between the positive and negative input vectors. After the model has been created, SVM classifies the test data to be on the negative or the positive side of that hyperplane.

In a recent paper, Hertel et al [53] used SVM to detect snoRNA without using information on the target ribosomal sequence with whom the snoRNA is supposed to interact. Our SVM is quite similar to the approach described in Hertel et al. but few differences should be noted.

SnoReport used a set of random sequences as negative examples, whereas in our SVM the negative training set contains of sequences that have all H/ACA structural features, but were checked experimentally not to be H/ACA-like molecules. The use of negative training set that is very similar to the positive training set increase the resolving power of the SVM method.

Also, the parsing of predicted secondary structure for each candidate in our report is more detailed than was used in snoReport. We parsed the hairpin of the H/ACA secondary structure to the eight features whereas in snoReport each hairpin of H/ACA molecule was parsed to the three features.

However, the SVM described in Hertel et al. is not specific to H/ACA like molecules in trypanosomes and is a stand alone program that is not part of a pipeline. Thus, it is not surprising that the authors noted that SnoReport was not able to detect any of the known H/ACA molecules in *L. major*.

### The features used by the SVM

The positive group of the training set was the set of the 34 known H/ACA-like molecules from *T. brucei *and the three molecules from the previous stage that were proved to be H/ACA snoRNAs (total of 37 molecules), and the negative group was a set of the 21 molecules that were suggested as H/ACA-like candidates by the previous stages of the screen but turned out experimentally not to be H/ACA RNAs (the 25 selected minus the four that were shown to be expressed). These molecules passed all the three stages of the pipeline but experimentally were still shown to not be expressed. Note that the use of such "false positive" sequences is important, as such a training set enables the machine learning algorithms to refine their model and correctly identify true sequences from the background of many false sequences. The test input group for SVM consisted of the 625 final candidates from T. brucei derived from the previous steps (excluding the sequences that were already analyzed by primer extension assay).

Each sequence used by SVM was transformed into a vector of the following 9 numerical features: The minimal free energy of the structure, the length of stem I, the existence of bulge in stem I (a binary feature), the size of loop I 5' to the pseudouridylation site and the size of loop I 3' to the pseudouridylation site, the number of bases complimentary to the ribosome (both the 5' and 3' to the pseudouridylation site), the size of stem II, and the size of the apical loop.

### Ten-fold cross validation test

Molecules in both the positive and the negative training groups possess basic H/ACA-like characteristics and therefore cannot be separated neither by the filters used in the deterministic screen nor by manual inspection.

As described above, the 37 sequences of the H/ACA-like snoRNAs were used as a positive training set and the 21 sequences of the false candidates were used as a negative training set in a ten-cross validation test. Note that as real H/ACA-like and negative sequences may have several predicted secondary structures and every predicted secondary structure is converted to a separate input vector for the SVM, the complete training set for the SVM consisted of 44 positive vectors, representing 37 different H/ACA-like sequences, and 79 negative vectors, which represented 21 different sequences.

In the ten-fold cross validation the positive and negative training data were randomly grouped into ten subsets of approximately equal size (each positive set contained 4 or 5 positive vectors and each negative set contained 7 or 8 negative vectors). Then, nine subsets of the positive group and nine subsets of the negative group were used as training data and the remaining two subsets constitute the test set.

This validation process was run one hundred times, each time the data were newly grouped into random ten subsets. The average accuracy of one hundred processes was calculated according to the formula (see Methods). The machine learning approach succeeded in distinguishing the members of the positive group from the members of the negative group with accuracy of 77.4%.

### SVM classification of the test data set

After calibration of the SVM on the training set with cross validation, we re-trained the SVM on the full training set that includes the 37 known H/ACA-like sequences which produce 44 positive vectors for the positive training set and the 21 sequences of the false candidates which produce 79 negative vectors for the negative training set.

**Figure 5:**
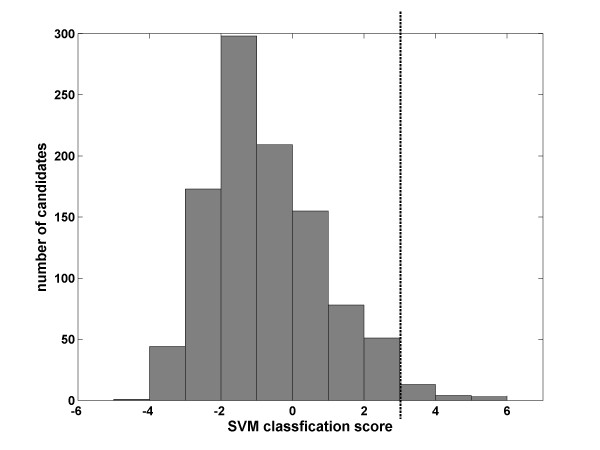
**The histogram plot of the SVM classification**. Histogram showing the number of candidate structures for each SVM score. Molecules with score higher than 3 (the dashed line) were subjected to experimental verification.

The trained SVM was then used to classify the 625 test sequences. The 625 candidate sequences were converted to 1029 vectors which were classified as either positive or negative. In our case the SVM classifier ranked every vector of the test data with score ranging from -6 to +6 (see Fig 5) reflecting its distance from the separation plane between the false and true candidates (for more details see Methods).

The positive test group (vectors that received score from 0 to 6) that consists of the vectors which are the most similar to the known H/ACA-like molecules comprised 304 vectors (179 sequences). In this group 13 out of the 179 sequences are known H/ACA-like molecules yielding an enrichment factor of 963.3.

Practically, 173 sequences are still too many for experimental validation. Thus, we chose a cutoff score of +3 and were left with 43 vectors representing 28 different candidate sequences classified as positive with the greatest confidence. 9 of these molecules were known H/ACA molecules reaching our final EF of 4263.3.

### Primer extension validation of the final results

**Figure 6:**
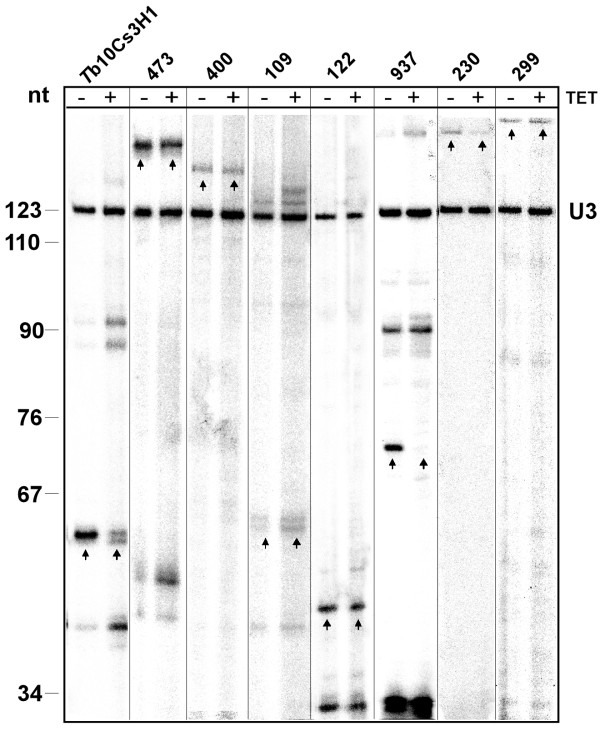
**Expression of the putative H/ACA-like RNA from the final list of the candidates**. RNA was subjected to primer extension with the oligonucleotides listed in the Additional File 2. Total RNA was extracted from cells before induction of the silencing of CBF5 (-Tet) or after 2 days of silencing (+Tet), as previously described (Barth et al., 2005). The extension products were separated on a 6% polyacrylamide 7 M urea gel next to labeled a marker (pBR322 MspI digest). The size of the marker in nt is indicated. The lanes for the different candidates are marked by their identification number. To control for equal loading of samples, the RNA was extended with oligonucleotide complementary to U3 snoRNA. The relevant extension products are marked with arrows.

As 9 out of the 28 chosen candidates are known H/ACA-like molecules, the remaining 19 candidates [see Additional File 5 for the list of the sequences] were examined for expression by primer extension, followed by analysis in CBF5 knockdown cells. Using primer extension analysis, 7 out of 19 candidates were found to be expressed as stable ncRNA molecules (see Fig 6). Unexpectedly, in several cases the lengths of the molecules were different from the ones predicted. For example, for candidates 473, 400, 230 and 299, the lengths of the primer extension products were about 150 nt. Candidate 473 had two extension products of about 150 nt and 50 nt in length; the latter is most probably a degradation product. Candidates 937, 122 and 109 are of the expected length.

In order to confirm the function of the 7 expressed RNAs, primer extension analysis was performed using RNA extracted from CBF5 silenced cells (+Tet) in comparison to uninduced cells (-Tet). Two of the seven molecules were shown to be H/ACA-like snoRNA, and the function of the remaining five molecules (as well as the function of one molecule from the intermediate validation step described above) remains unknown, as they were insensitive to CBF5 silencing.

In attempt to explore the potential function of these six ncRNA, we performed homology search of these sequences against the RFAM database [68] using BLAST [60], but no potential homologs were found; therefore additional laboratory experiments must be performed in order to reveal the function of these six ncRNAs.

### Manual exploration of clusters

**Figure 7:**
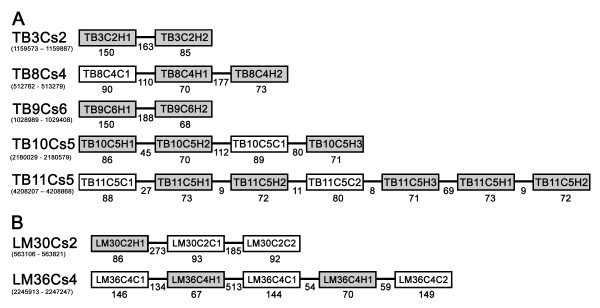
**The schematic structure and location of the predicted clusters**. (A) Four novel snoRNA clusters detected in this study in *T. brucei*. (B) Two clusters detected in *L. major*. The names of each molecule in the cluster are given according to snoRNA nomenclature in *T. brucei *and *L. major *(TB or LM; chromosome number; C, cluster number; C, C/D number or H, H/ACA-like number). The C/D snoRNAs are shown as lightly-shaded boxes, whereas the H/ACA-like RNAs are in dark boxes. Thinner lines indicate an intergenic region, with size indicated below the line. The lengths of snoRNA genes (± 3 bp) are indicated below the box. The small numbers below the name of each cluster indicates the position of the cluster in the genome database of *T. brucei *GD release 4 [57], and *L. major *genome release 5 [58].

Because of the fact that most, if not all, snoRNAs in trypanosomatids are arranged in clusters, we performed bioinformatic analysis of the genomic regions surrounding five of the verified H/ACA-like snoRNAs [three from the first stage of PsiScan (before the SVM) and two from the last stage]. We found indications for potential clusters in all cases (see Fig 7-A). Note that during the manual exploration of clusters in genomic loci of a new validated H/ACA-like molecules we allowed more relaxed parameters than those described in the consensus structure (see Fig. 1) because of two reasons: First, the regions of searching for a new molecules was short (about 1000 nt each) and there were fewer false results to deal with. Second, the consensus structure was developed based on the known H/ACA-like molecules identified to date and we wanted to try to find new functional H/ACA-like snoRNAs that may not fully adhere to the current consensus structure. Therefore, we increased the range of sizes for the stem I and the pseudouridylation pocket, and enabled the existence of one bulge in the stem I.

**Figure 8:**
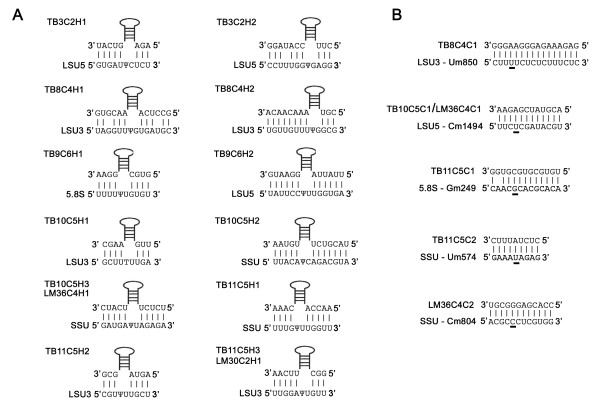
**The predicted targets on the rRNA for H/ACA-like and C/D snoRNAs based on sequence complementarity**. (A) The potential targets for H/ACA-like molecules. The name of the snoRNA and the region of complementarity on rRNAs are given. (SSU) Ribosomal RNA small subunit; (LSU5 and LSU3) ribosomal RNA large subunit, 5' and 3' half, respectively; (5.8S) 5.8S ribosomal RNA. Pseudouridines are marked by ψ. (B) The potential targets for C/D snoRNA molecules. The designation of rRNA is as described in A. The 2'-O-methylation sites are underlined and the positions on rRNA are given.

The first cluster is located on chromosome 3 and consists of two H/ACA-like ncRNAs, including one that is unusually long (about 150 nt). This cluster is the first one observed to include only H/ACA-like molecules. The second cluster is located on chromosome 8 and consists of two H/ACA-like ncRNAs and one C/D. The third is located on chromosome 9 and consists of two H/ACA-like snoRNAs. As in first case, this cluster includes only H/ACA-like molecules, and the first H/ACA-like molecule is about 150 nt in length. The fourth cluster is located on chromosome 10 and consists of three H/ACA-like RNAs and one C/D. The fifth is located on chromosome 11 and consists of three H/ACA-like RNAs and two C/Ds. Note that this cluster contains partial tandem repeat thus TB11C5H1 TB11C5H2 appear twice. The potential targets on the rRNA for each new H/ACA-like and C/D molecules were predicted (see Fig 8). Note that the genomic organization of these clusters is different from the clusters we described before [33], because the previously described clusters contain more C/D than H/ACA-like molecules.

Indeed, several molecules that were found by manual exploration are different from the consensus structure described in Figure 1. For example, in the molecule TB3C2H2, an upstream side of pseudouridylation pocket is 15 nt long, whereas the maximum size in consensus structure is 11 nt.

Another example is TB8C4H1, which stem I is of size 7 bp, but contains bulge that is not allowed by the consensus structure.

In addition, we found that two out of five verified H/ACA-like molecules have orthologs in *L. major*. The first one (LM30C2H1) is novel H/ACA-like molecule, which is located near two previously described C/D molecules (see Fig 7-B). The second ortholog is LM36C4H1, which has two inexact repeats in the *L. major *genome (see Fig 7-B); however the pseudouridylation pocket in both cases is perfectly conserved (see Fig 8).

Two novel C/D snoRNAs were found in the genomic locus of LM36C4H1. The first C/D is LM36C4C1, with two inexact repeats in the *L. major *genome; this RNA species guides a methylation on the same site as TB10C5C1 (see Fig 8). The second C/D snoRNA (LM36C4C2) has no ortholog in *T. brucei *and is encoded by a unique gene. In conclusion, this study expands the collection of snoRNAs in trypanosomes by 14 novel H/ACA and 6 novel C/D molecules [see Additional File 6 for the list of the sequences].

## Discussion

The current estimate of the number of different methylation sites (Nms) in trypanosomes is 110–130 Nm, based on partial mapping and on the 120 Nms identified in the study of Crithidia [39]. In other eukaryotes, the number of Nms and pseudouridines (Ψs) are similar to each other. However, trypanosomes may have more Nms than Ψs because the Nms stabilize the rRNA and are essential for preserving ribosome function during cycling of the parasite from the insect to the mammalian host [38]. In addition, all the snoRNA clusters described previously carried a mixture of both C/D and H/ACA-like RNAs [32,33] and usually contained twice as many C/D as H/ACA-like genes. Based on these two observations, we assumed that the trypanosome genome may contain fewer H/ACA than C/D molecules. Thus, since there are about 130 Nm in *T. brucei*, and since most C/D molecules can each guide two modifications, we estimate that trypanosomes may have 70–80 C/Ds and less than 70 H/ACA-like molecules.

However, in this study, we identified for the first time two small clusters (TB3Cs2 and TB9C6) which consist of only H/ACA-like molecules (Fig 7-A). This new finding undermines the assumption of the number of H/ACA-like molecules. One intriguing possibility is that there are many more H/ACA-like RNAs "hiding" in the genome in clusters carrying only H/ACA-like molecules. Therefore, based on our findings on H/ACA-only clusters, we propose that the number of pseudouridylation sites may be as large as in other eukaryotes e.g. more than 100 in humans [69].

One of the most interesting findings emerging from our screen was the finding of several novel small RNAs that share many structural features with H/ACA-like RNA and are conserved in trypanosome species but are not destabilized in CBF5 knock down cells. We call these molecules AGA-like. These molecules may be non-coding RNAs that are not related to guide RNAs from the AGA family. Since these molecules have, by our search criteria, complementarity to rRNA, we suggest that they constitute another group of ncRNA that may also function in rRNA processing.

We recently reported the existence of 124 Nms in *T. brucei *[38], but failed to identify the complete C/D snoRNAs that guide these modifications. We suggested that trypanosome may have an additional family of guide RNAs with a similar function. The AGA-like RNA may represent such a family of RNAs that also function in rRNA processing, but bind a different subset of RNP proteins.

We also note that several of these RNAs are larger in size than the conventional H/ACA-like RNAs, which range in size from 70–90 bases. Note that other classes of H/ACA-like RNAs such as scaRNA RNAs, which contain both C/D and H/ACA RNA domains and guide modifications on snRNAs [10] were not yet identified in trypanosomes. In addition, telomerase RNA, which is also an H/ACA RNA in different eukaryotes, was also not identified in any of the trypanosomatid species. We are currently examining whether the novel molecules that we have described in this study might constitute the trypanosome homologs of these currently missing ncRNAs. Finally, we can not totally rule out the possibility that these RNAs are regular H/ACA-like molecules but they very stable compared to the guide RNA we examined previously, and thus their destabilization following silencing is less evident [56].

Recently, it was suggested that contrary to previous belief, very large fractions of eukaryotic genomes are expressed [70-73], and there is an on-going debate about the biological significance of these transcripts. This debate has practical implications for our studies, since we wished to show that the large fraction of the selected candidate molecules that are expressed is due to the predictive ability of the algorithm, rather than due to a high level of background expression.

**Figure 9:**
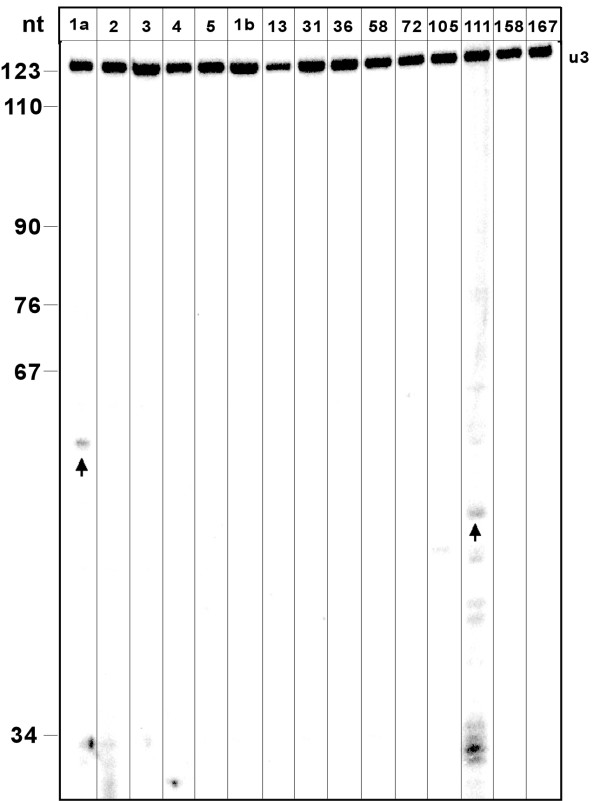
**The presence of ncRNA in random sequences**. RNA was subjected to primer extension with the oligonucleotides listed in the Additional File 2. The extension products were separated on a 6% polyacrylamide 7 M urea gel next to a labeled marker (pBR322 MspI digest). The size of the marker in nt is indicated. The lanes for the different candidates are marked by their identification number. To control for equal loading, the RNA was extended with oligonucleotide complementary to U3 snoRNA. The relevant extension products are marked with arrows.

Thus, we decided to see if fragments chosen at random from the *T. brucei *genome encode for small RNAs. Towards this goal we performed an additional primer extension analysis from 15 randomly chosen sequences [see Additional File 7 for the list of the sequences]. Five out of 15 sequences of 60–80 nt length were selected at random from intergenic regions of the *T. brucei *genome. The remaing 10 sequences were chosen randomly from the (approximately 1 million) sequences that passed the first stage of the pipeline of our method – the genome wide search for sequences that fit the basic constraints of H/ACA-like molecules. We validated that none of these 10 sequences are annotated as functional regions of any form in the *T. brucei *genome. Two sequences out of the 15 were expressed (see Fig 9).

Notwithstanding the small sample size of this analysis, we note that short sequences chosen at random from regions that are not known to have any function in the genome of *T. brucei *still contain expressed ncRNA (2 out of 15). The small sample size does not allow numerical extrapolation, but is consistent with the possibility of thousands of ncRNA expressed genome-wide [70].

It is clear that our procedure is successful in enriching the fraction of molecules that are expressed from 2 out of 15 (13%) in the random assay to 7 out of 19 (36%) in our final validation step, in additional to 9 known H/ACA-like molecules that have been retrieved.

SnoGPS [51] is a program that was designed to search for the classic double hairpin H/ACA molecules in eukaryotic genomes. However, it can be configured to run in single-stem mode as well. To compare the performance of SnoGPS to our Psiscan pipeline, we have only included those results that terminate with an AGA box. Using its default values SnoGPS returned 11,886,827 hits that we merged to represent 310,413 sequences. In this ensemble 32 out of the 34 known H/ACA-like in *T. Brucei *were found. Thus, the performance of SnoGPS is comparable to the first stage of our pipeline where we ended up with 199,073 sequences that contain 32 of the known molecules. SnoGPS does not provide a mechanism to narrow down the large number of candidates; however it is possible to use the scores that SnoGPS produces for each hit to rank the candidates. Only three known H/ACA molecules were found in the top 28 candidates of SnoGPS. As we mentioned above, the 28 final candidates that Psiscan produced contained 9 of the known molecules. Thus, our procedure suggests a significant improvement over using SnoGPS alone.

The nine known H/ACA-like molecules detected by Psiscan in the final stage represent about 25% of the previously known repertoire of 34 H/ACA-like molecules in *T. brucei*. Such a detection level of known molecules is reasonable, as relaxing some of the constraints along the pipeline may lead to a larger number of known molecules recovered, but at the cost of increasing the number of false positives in the predicted candidates.

Furthermore, as more data become available from the experimental validation of our current set of candidates, the SVM approach will enable us to further refine and improve the performance of the method to detect additional, still missing, H/ACA molecules in Trypanosomes.

## Conclusion

In this study we developed Psiscan, a new computational method for identification of H/ACA-like snoRNAs in trypanosomatids. Identification of new double hairpin H/ACA molecules in mammalian genomes is a difficult bioinformatic challenge because of the short conserved motifs and rRNA recognition sequences. The identification of single hairpin H/ACA-like snoRNAs is even harder. As trypanosome H/ACA molecules are different from the canonical box H/ACA snoRNAs of yeast, vertebrates and Archaea it is not surprising that the computational approaches for identification of single hairpin H/ACA that were published to date failed to identify H/ACA-like snoRNAs in trypanosome species.

However, while the signals for detecting H/ACA-like molecules in trypanosomes are weak, they do exist. Thus, a careful combination of methods can be used to search for additional such molecules. Our single hairpin H/ACA finder in trypanosomes, Psiscan, consists of deterministic genome wide search for sequences carries H/ACA-like properties followed by a machine learning ranking. We applied our method on three related genomes: *T. brucei*, *T. cruzi *and *L. major *and verified the results from *T. brucei *by primer extension assays. Eleven new ncRNA molecules were discovered including five new H/ACA-like RNAs that were not described before, and six novel RNAs with unknown function. Bioinformatic analysis of the genomic loci of five validated H/ACA-like snoRNAs led to the prediction of five new clusters which contain an additional seven new H/ACA-like molecules and four new C/D snoRNAs. Bioinformatic analysis revealed that two of these clusters also exist in *L. major *(See Fig. 7-B). All together, this study increased our repertoire by 14 H/ACA-like and eight C/D snoRNAs molecules.

This study suggests that we are at just the tip of the iceberg in unraveling the large number of ncRNA families in trypanosomes, and the large variety within these families. The work presented here advocates for combining computational approaches with experimental studies to advance our knowledge of this exciting new world of ncRNAs.

## Authors' contributions

IM characterized the H/ACA molecules and implemented the pipeline and the SVM computations. TD did the bioinformatic studies involved in this study. YH devised the algorithmic tools used in the computational pipeline. AH performed the mutation analysis on the H/ACA RNAs. RH performed the primer extension to validate the predicted RNAs and whether these belong to the H/ACA group. YZ performed together with AH the mutation analysis on the H/ACA. SM suggested the experimental strategies to validate the RNA as H/ACA and designed the mutation analysis. RU suggested the bioinformatic approach used in this research and oversaw its execution.

IM, SM and RU wrote the manuscript.

## Supplementary Material

Additional file 1 **List of the oligos specific to L. collosoma.** The oligos were used as primers for tagging h2 H/ACA by PCR mutagenesis. Click here for file

Additional file 2 **List of the oligos specific to *T. brucei *used by primer extension analysis.** The oligos were used for analysis of expression of potential H/ACA-like sequences predicted by Psiscan and analysis of expression of the random sequences. Click here for file

Additional file 3 **Constrains used for MFOLD program for H/ACA-like molecules predicted by Psiscan.** Examples of user-defined constraints for MFOLD program for H/ACA-like molecules. Our constraints prevent base pairing of the sequences which recognize the rRNA in the pseudouridylation pocket and force two nucleotides from stem II immediately after the pseudouridylation pocket to be base paired. Click here for file

Additional file 4 **List of the sequences of intermediate results.** Intermediate results consist of 25 sequences that were checked for H/ACA-like expression by primer extension analysis. Click here for file

Additional file 5 **List of the sequences of final results.** Final results consist of 19 sequences that were checked for H/ACA-like expression by primer extension analysis. Click here for file

Additional file 6 **List of the novel predicted H/ACA-like and C/D snoRNAs.** The list consists of the novel H/ACA-like snoRNAs predicted by Psiscan method and of novel H/ACA-like and C/D snoRNAs predicted by manual exploration of clusters in *T. brucei *and *L. major*. Click here for file

Additional file 7 **List of the random sequences that were analyzed by primer extension. **The list consists of 15 sequences that were randomly selected from intergenic regions of the *T. brucei *genome. Click here for file

## References

[B1] Dennis PP, Omer A, Lowe T (2001). A guided tour: small RNA function in Archaea. Mol Microbiol.

[B2] Omer AD, Lowe TM, Russell AG, Ebhardt H, Eddy SR, Dennis PP (2000). Homologs of small nucleolar RNAs in Archaea. Science.

[B3] Filipowicz W, Pogacic V (2002). Biogenesis of small nucleolar ribonucleoproteins. Curr Opin Cell Biol.

[B4] Kiss T (2002). Small nucleolar RNAs: an abundant group of noncoding RNAs with diverse cellular functions. Cell.

[B5] Tollervey D, Kiss T (1997). Function and synthesis of small nucleolar RNAs. Curr Opin Cell Biol.

[B6] Decatur WA, Fournier MJ (2003). RNA-guided nucleotide modification of ribosomal and other RNAs. J Biol Chem.

[B7] Ganot P, Bortolin ML, Kiss T (1997). Site-specific pseudouridine formation in preribosomal RNA is guided by small nucleolar RNAs. Cell.

[B8] Kiss-Laszlo Z, Henry Y, Bachellerie JP, Caizergues-Ferrer M, Kiss T (1996). Site-specific ribose methylation of preribosomal RNA: a novel function for small nucleolar RNAs. Cell.

[B9] Ni J, Tien AL, Fournier MJ (1997). Small nucleolar RNAs direct site-specific synthesis of pseudouridine in ribosomal RNA. Cell.

[B10] Darzacq X, Jady BE, Verheggen C, Kiss AM, Bertrand E, Kiss T (2002). Cajal body-specific small nuclear RNAs: a novel class of 2'-O-methylation and pseudouridylation guide RNAs. Embo J.

[B11] Uliel S, Liang XH, Unger R, Michaeli S (2004). Small nucleolar RNAs that guide modification in trypanosomatids: repertoire, targets, genome organisation, and unique functions. Int J Parasitol.

[B12] Kishore S, Stamm S (2006). The snoRNA HBII-52 regulates alternative splicing of the serotonin receptor 2C. Science.

[B13] Yu YT, Terns RM, Terns MP (2005). Fine-tuning of RNA Functions by Modification and Editing. Topics in Current Genetics.

[B14] Collins K (2006). The biogenesis and regulation of telomerase holoenzymes. Nat Rev Mol Cell Biol.

[B15] Bortolin ML, Ganot P, Kiss T (1999). Elements essential for accumulation and function of small nucleolar RNAs directing site-specific pseudouridylation of ribosomal RNAs. Embo J.

[B16] Bousquet-Antonelli C, Henry Y, G'Elugne JP, Caizergues-Ferrer M, Kiss T (1997). A small nucleolar RNP protein is required for pseudouridylation of eukaryotic ribosomal RNAs. Embo J.

[B17] Dragon F, Pogacic V, Filipowicz W (2000). In vitro assembly of human H/ACA small nucleolar RNPs reveals unique features of U17 and telomerase RNAs. Mol Cell Biol.

[B18] Henras A, Henry Y, Bousquet-Antonelli C, Noaillac-Depeyre J, Gelugne JP, Caizergues-Ferrer M (1998). Nhp2p and Nop10p are essential for the function of H/ACA snoRNPs. Embo J.

[B19] Lafontaine DL, Tollervey D (1998). Birth of the snoRNPs: the evolution of the modification-guide snoRNAs. Trends Biochem Sci.

[B20] Rozhdestvensky TS, Tang TH, Tchirkova IV, Brosius J, Bachellerie JP, Huttenhofer A (2003). Binding of L7Ae protein to the K-turn of archaeal snoRNAs: a shared RNA binding motif for C/D and H/ACA box snoRNAs in Archaea. Nucleic Acids Res.

[B21] Watanabe Y, Gray MW (2000). Evolutionary appearance of genes encoding proteins associated with box H/ACA snoRNAs: cbf5p in Euglena gracilis, an early diverging eukaryote, and candidate Gar1p and Nop10p homologs in archaebacteria. Nucleic Acids Res.

[B22] Watkins NJ, Gottschalk A, Neubauer G, Kastner B, Fabrizio P, Mann M, Luhrmann R (1998). Cbf5p, a potential pseudouridine synthase, and Nhp2p, a putative RNA-binding protein, are present together with Gar1p in all H BOX/ACA-motif snoRNPs and constitute a common bipartite structure. Rna.

[B23] Li L, Ye K (2006). Crystal structure of an H/ACA box ribonucleoprotein particle. Nature.

[B24] Sogin ML, Elwood HJ, Gunderson JH (1986). Evolutionary diversity of eukaryotic small-subunit rRNA genes. Proc Natl Acad Sci USA.

[B25] Liang XH, Haritan A, Uliel S, Michaeli S (2003). trans and cis splicing in trypanosomatids: mechanism, factors, and regulation. Eukaryot Cell.

[B26] Simpson L, Sbicego S, Aphasizhev R (2003). Uridine insertion/deletion RNA editing in trypanosome mitochondria: a complex business. Rna.

[B27] White TC, Rudenko G, Borst P (1986). Three small RNAs within the 10 kb trypanosome rRNA transcription unit are analogous to domain VII of other eukaryotic 28S rRNAs. Nucleic Acids Res.

[B28] Liang XH, Liu L, Michaeli S (2001). Identification of the first trypanosome H/ACA RNA that guides pseudouridine formation on rRNA. J Biol Chem.

[B29] Tang TH, Bachellerie JP, Rozhdestvensky T, Bortolin ML, Huber H, Drungowski M, Elge T, Brosius J, Huttenhofer A (2002). Identification of 86 candidates for small non-messenger RNAs from the archaeon Archaeoglobus fulgidus. Proc Natl Acad Sci USA.

[B30] Russell AG, Schnare MN, Gray MW (2004). Pseudouridine-guide RNAs and other Cbf5p-associated RNAs in Euglena gracilis. Rna.

[B31] Brown JW, Echeverria M, Qu LH (2003). Plant snoRNAs: functional evolution and new modes of gene expression. Trends Plant Sci.

[B32] Liang XH, Hury A, Hoze E, Uliel S, Myslyuk I, Apatoff A, Unger R, Michaeli S (2007). Genome-wide analysis of C/D and H/ACA-like small nucleolar RNAs in Leishmania major indicates conservation among trypanosomatids in the repertoire and in their rRNA targets. Eukaryot Cell.

[B33] Liang XH, Uliel S, Hury A, Barth S, Doniger T, Unger R, Michaeli S (2005). A genome-wide analysis of C/D and H/ACA-like small nucleolar RNAs in Trypanosoma brucei reveals a trypanosome-specific pattern of rRNA modification. Rna.

[B34] Liang XH, Liu Q, Michaeli S (2003). Small nucleolar RNA interference induced by antisense or double-stranded RNA in trypanosomatids. Proc Natl Acad Sci USA.

[B35] Xu Y, Liu L, Lopez-Estrano C, Michaeli S (2001). Expression studies on clustered trypanosomatid box C/D small nucleolar RNAs. J Biol Chem.

[B36] Dunbar DA, Chen AA, Wormsley S, Baserga SJ (2000). The genes for small nucleolar RNAs in Trypanosoma brucei are organized in clusters and are transcribed as a polycistronic RNA. Nucleic Acids Res.

[B37] Roberts TG, Sturm NR, Yee BK, Yu MC, Hartshorne T, Agabian N, Campbell DA (1998). Three small nucleolar RNAs identified from the spliced leader-associated RNA locus in kinetoplastid protozoans. Mol Cell Biol.

[B38] Barth S, Shalem B, Hury A, Tkacz ID, Liang XH, Uliel S, Myslyuk I, Doniger T, Salmon-Divon M, Unger R (2008). Elucidating the role of C/D snoRNA in rRNA processing and modification in Trypanosoma brucei. Eukaryot Cell.

[B39] Gray MW (1979). The ribosomal RNA of the trypanosomatid protozoan Crithidia fasciculata: physical characteristics and methylated sequences. Can J Biochem.

[B40] Russell AG, Schnare MN, Gray MW (2006). A large collection of compact box C/D snoRNAs and their isoforms in Euglena gracilis: structural, functional and evolutionary insights. J Mol Biol.

[B41] Torchet C, Badis G, Devaux F, Costanzo G, Werner M, Jacquier A (2005). The complete set of H/ACA snoRNAs that guide rRNA pseudouridylations in Saccharomyces cerevisiae. Rna.

[B42] Fedorov A, Stombaugh J, Harr MW, Yu S, Nasalean L, Shepelev V (2005). Computer identification of snoRNA genes using a Mammalian Orthologous Intron Database. Nucleic Acids Res.

[B43] Gaspin C, Cavaille J, Erauso G, Bachellerie JP (2000). Archaeal homologs of eukaryotic methylation guide small nucleolar RNAs: lessons from the Pyrococcus genomes. J Mol Biol.

[B44] Lowe TM, Eddy SR (1999). A computational screen for methylation guide snoRNAs in yeast. Science.

[B45] Schattner P, Barberan-Soler S, Lowe TM (2006). A computational screen for mammalian pseudouridylation guide H/ACA RNAs. Rna.

[B46] Brown JW, Clark GP, Leader DJ, Simpson CG, Lowe T (2001). Multiple snoRNA gene clusters from Arabidopsis. Rna.

[B47] Barneche F, Gaspin C, Guyot R, Echeverria M (2001). Identification of 66 box C/D snoRNAs in Arabidopsis thaliana: extensive gene duplications generated multiple isoforms predicting new ribosomal RNA 2'-O-methylation sites. J Mol Biol.

[B48] Qu LH, Meng Q, Zhou H, Chen YQ (2001). Identification of 10 novel snoRNA gene clusters from Arabidopsis thaliana. Nucleic Acids Res.

[B49] Edvardsson S, Gardner PP, Poole AM, Hendy MD, Penny D, Moulton V (2003). A search for H/ACA snoRNAs in yeast using MFE secondary structure prediction. Bioinformatics.

[B50] Yang JH, Zhang XC, Huang ZP, Zhou H, Huang MB, Zhang S, Chen YQ, Qu LH (2006). snoSeeker: an advanced computational package for screening of guide and orphan snoRNA genes in the human genome. Nucleic Acids Res.

[B51] Schattner P, Decatur WA, Davis CA, Ares M, Fournier MJ, Lowe TM (2004). Genome-wide searching for pseudouridylation guide snoRNAs: analysis of the Saccharomyces cerevisiae genome. Nucleic Acids Res.

[B52] Hertel J, Hofacker IL, Stadler PF (2007). SnoReport: Computational identification of snoRNAs with unknown targets. Bioinformatics.

[B53] Hertel J, Hofacker IL, Stadler PF (2008). SnoReport: computational identification of snoRNAs with unknown targets. Bioinformatics.

[B54] Muller S, Charpentier B, Branlant C, Leclerc F (2007). A dedicated computational approach for the identification of archaeal H/ACA sRNAs. Methods Enzymol.

[B55] Muller S, Leclerc F, Behm-Ansmant I, Fourmann JB, Charpentier B, Branlant C (2008). Combined in silico and experimental identification of the Pyrococcus abyssi H/ACA sRNAs and their target sites in ribosomal RNAs. Nucleic Acids Res.

[B56] Barth S, Hury A, Liang X-h, Michaeli S (2005). Elucidating the Role of H/ACA-like RNAs in trans-Splicing and rRNA Processing via RNA Interference Silencing of the Trypanosoma brucei CBF5 Pseudouridine Synthase. THE JOURNAL OF BIOLOGICAL CHEMISTRY.

[B57] Berriman M, Ghedin E, Hertz-Fowler C, Blandin G, Renauld H, Bartholomeu DC, Lennard NJ, Caler E, Hamlin NE, Haas B (2005). The genome of the African trypanosome Trypanosoma brucei. Science.

[B58] Ivens AC, Peacock CS, Worthey EA, Murphy L, Aggarwal G, Berriman M, Sisk E, Rajandream MA, Adlem E, Aert R (2005). The genome of the kinetoplastid parasite, Leishmania major. Science.

[B59] El-Sayed NM, Myler PJ, Blandin G, Berriman M, Crabtree J, Aggarwal G, Caler E, Renauld H, Worthey EA, Hertz-Fowler C (2005). Comparative genomics of trypanosomatid parasitic protozoa. Science.

[B60] Altschul SF, Gish W, Miller W, Myers EW, Lipman DJ (1990). Basic local alignment search tool. J Mol Biol.

[B61] Zuker M (2003). Mfold web server for nucleic acid folding and hybridization prediction. Nucleic Acids Res.

[B62] Yang ZR (2004). Biological applications of support vector machines. Brief Bioinform.

[B63] Vapnik VN (1995). The Nature of Statistical Learning Theory. Springer-Velgrad.

[B64] Joachims T (1999). Making large-Scale SVM Learning Practical. Advances in Kernel Methods – Support Vector Learning.

[B65] Glodring A, Karchi M, Michaeli S (1995). The spliced leader RNA gene of Leptomonas collosoma. Exp Parasitol.

[B66] Liang XH, Ochaion A, Xu YX, Liu Q, Michaeli S (2004). Small nucleolar RNA clusters in trypanosomatid Leptomonas collosoma. Genome organization, expression studies, and the potential role of sequences present upstream from the first repeated cluster. J Biol Chem.

[B67] Horesh Y, Doniger T, Michaeli S, Unger R (2007). RNAspa: a shortest path approach for comparative prediction of the secondary structure of ncRNA molecules. BMC Bioinformatics.

[B68] Griffiths-Jones S, Moxon S, Marshall M, Khanna A, Eddy SR, Bateman A (2005). Rfam: annotating non-coding RNAs in complete genomes. Nucleic Acids Res.

[B69] Maden BE, Corbett ME, Heeney PA, Pugh K, Ajuh PM (1995). Classical and novel approaches to the detection and localization of the numerous modified nucleotides in eukaryotic ribosomal RNA. Biochimie.

[B70] Mattick JS, Makunin IV (2006). Non-coding RNA. Hum Mol Genet.

[B71] Carninci P, Kasukawa T, Katayama S, Gough J, Frith MC, Maeda N, Oyama R, Ravasi T, Lenhard B, Wells C (2005). The transcriptional landscape of the mammalian genome. Science.

[B72] Cheng J, Kapranov P, Drenkow J, Dike S, Brubaker S, Patel S, Long J, Stern D, Tammana H, Helt G (2005). Transcriptional maps of 10 human chromosomes at 5-nucleotide resolution. Science.

[B73] Birney E, Stamatoyannopoulos JA, Dutta A, Guigo R, Gingeras TR, Margulies EH, Weng Z, Snyder M, Dermitzakis ET, Thurman RE (2007). Identification and analysis of functional elements in 1% of the human genome by the ENCODE pilot project. Nature.

