# Supplementary material for: Psiscan: a computational approach to identify H/ACA-like and AGA-like non-coding RNA in trypanosomatid genomes
Source: BMC Bioinformatics. 2008 Nov 5;9:471. doi: 10.1186/1471-2105-9-471 (PMC2613932; doi:10.1186/1471-2105-9-471)
Supplement: Additional file 1 — List of the oligos specific to L. collosoma. The oligos were used as primers for tagging h2 H/ACA by PCR mutagenesis. [file 1471-2105-9-471-S1.doc]

**List of the oligos specific to *L.collosoma*.**

The oligos were used as primers for tagging h2 H/ACA by PCR mutagenesis.

-5677, 5`-CACGAATCTACAGGAATTCTCGAGACTGTGCAA-3`,antisense, from positions 17-43 of h2 snoRNA coding region, carrying insertion of *EcoR*I site between position 26-27, used for creation of Tag1-h2 snoRNA.

*-5678*, 5`-GTCCCGTGCACGGAATTCAATCTACAGTCGAGAC-3`, antisense, from positions 24-51 of h2 snoRNA coding region, carrying insertion of *EcoR*I site in position 39-40, used for creation of Tag2-h2 snoRNA.

*-8903,* 5`-CCGTGCACGAATTACTACAGTCGAGATACTGTGCAACCT-3`, antisense, from positions 14-48 of h2 snoRNA coding region, carrying insertion of two nucleotides between position 37-38 and another two nucleotides between positions 24-25 (underlined), used for creation of Tag3-h2 snoRNA.

*-4207*, 5-`CACAGCGGGGAAAGCCAGAATTGTCCCG-3`, antisense, from positions 46-73 of h2 snoRNA coding carrying substitution of the AGA box in position 61-63 to TTT (underlined), used for creation of the M4 mutated h2 snoRNA.

*-4208*, 5-`CACAGCGGGGTGTGCCAGAATTGTCCCG-3`, antisense, from positions 46-73 of h2 snoRNA coding region, carrying substitution of the AGA box in position 61-63 to ACA (underlined), used for creation of the M5 mutated h2 snoRNA.

*-4209*, 5-`CACAGCGGGGTTTGCCAGAATTGTCCCG-3`, antisense, from positions 46-73 of h2 snoRNA coding region, carrying substitution of the AGA box in position 61-63 to AAA (underlined), used for creation of the M6 mutated h2 snoRNA.

*-9324,*  5`-GGTCTGCCAGTTTTGTCCCGTGC-3`, antisense, from positions 43-65 of h2 snoRNA coding region, carrying substitution of two nucleotides in position 54-55 (underlined), used for creation of the M7 mutated h2 snoRNA.

*-9325*, 5`-CTGCCAGAATCAACCCGTGCAC -3`, antisense, from positions 41-62 of h2 snoRNA coding region, carrying substitution of two nucleotides in position 50-51 (underlined), used for creation of the M8 mutated h2 snoRNA.

- *9326,* 5`- GAATTGTCCCCAGCACGAATC-3`*,* antisense, from positions 36-56 of h2 snoRNA coding region, carrying substitution of two nucleotides in position 45-46 (underlined), used for creation of the M9 mutated h2 snoRNA.

*-7500,* 5`-CGCTCTAGAATTCGAACCCCATCGCACGA-3`, anti sense, from positions 85-100 of h2 snoRNA coding region, barring *Xba*I site (underlined), used for amplification and cloning of the mutated h2 and snoRNA2 in to pX-neo vector.

*-24355*, 5'-CGGGATCCTTGTGCGTCGACGTGCAGT-3', sense, from positions -196 to -178 of the coding region barring *BamH*I site (underlined), used for amplification and cloning of the mutated h2 and snoRNA2 in to pX-neo vector.
