# Supplementary material for: Psiscan: a computational approach to identify H/ACA-like and AGA-like non-coding RNA in trypanosomatid genomes
Source: BMC Bioinformatics. 2008 Nov 5;9:471. doi: 10.1186/1471-2105-9-471 (PMC2613932; doi:10.1186/1471-2105-9-471)
Supplement: Additional file 2 — List of the oligos specific to T. brucei used by primer extension analysis. The oligos were used for analysis of expression of potential H/ACA-like sequences predicted by Psiscan and analysis of expression of the random sequences. [file 1471-2105-9-471-S2.doc]

**List of the oligos specific to *T.brucei* used by primer extension analysis.**

The oligos were used for analysis of expression of potential H/ACA-like sequences predicted by Psiscan and analysis of expression of the random sequences.

*6524,* 5' tgccgttcatcgaac '3 *,* antisense*,* from position 109-123 of U3 control.

*8160* ,5' AAGGCTGTGCACGGTTTC '3 , antisense, from position 53-70 of *Tb10Cs3H1.*

*3066*, 5`-TGCCGCTCCAAATGCC -3`, antisense, from positions 49-64 of psiscan putative candidate no. 473.

*3061*, 5`-ACGCACACAAACACCCAC -3`, antisense, from positions 41-58 of psiscan putative candidate no. 400.

*3062*, 5`-TTCCCCACCTTCTCCA -3`,antisense, from positions 52-67 of psiscan putative candidate no. 109.

*3060*, 5`-CCTTCCCTTCCTCCTCTCTT -3`, antisense, from positions 47-66 of psiscan putative candidate no. 122.

*3374*, 5`-GAAGGTGTTGTTTCGGTTT -3`, antisense, from positions 47-65 of psiscan putative candidate no. 937.

*3370*, 5`-CACACTTCAAAGCATTAGCA -3`, antisense, from positions 31-50 of psiscan putative candidate no. 230.

*3065*, 5`-GAAGACGCCCCACGCA -3`, antisense, from positions 56-71 of psiscan putative candidate no. 299.

*7825*, 5`-CTCTCTTGGAGAACTTACAA -3`, antisense, for random sequence 1a.

*9462*, 5`- atcaatcagttgtttatttt -3`, antisense, for random sequence 2.

*9464*, 5`- gtttgtttgttgattctg -3`, antisense, for random sequence 3.

*9466*, 5`- taagacccttcaggacag -3`, antisense, for random sequence 4.

*9486*, 5`- tttatatcttctcttgcatft -3`, antisense, for random sequence 5.

*9459*, 5`- gaagcfftacctctcatt -3`, antisense, for random sequence 1b.

*7809*, 5`-AGATCTGTGCAGAAAACTAA -3`, antisense, for random sequence 13.

*7813*, 5`- AGCTCTTCTACACTTTGA -3`, antisense, for random sequence 31.

*7827* 5`- ACTTCTTTGAACACAACTCC -3`, antisense, for random sequence 36.

*7821*, 5`- ATCTTTTTCCAACCGAGAAC -3`, antisense, for random sequence 58.

*7823*, 5`- CGTAGACGTCCGAAGTAGTG -3`, antisense, for random sequence 72.

*7810*, 5`-TCTTCTCTAACGCTACATTT -3`, antisense, for random sequence 105.

*7817*, 5`- TCATCTCACGTCTCTTTCTA -3`, antisense, for random sequence 111.

*7815*, 5`- TTCTGCGGCACACGGTTA -3`, antisense, for random sequence 158.

*7819*, 5`-TGGTCTGCAAGCAGTAACTC -3`, antisense, for random sequence 167.

5' – GCTTTTGAACCACATTGCAC – '3 , antisense, from position 40-59 of psiscan intermediate algorithm candidate no. 16.

5' – GAGGGCAATGACACTGGGAA – '3 , antisense, from position 44-63 of Psiscan intermediate candidate no. 25.
