# Supplementary material for: Psiscan: a computational approach to identify H/ACA-like and AGA-like non-coding RNA in trypanosomatid genomes
Source: BMC Bioinformatics. 2008 Nov 5;9:471. doi: 10.1186/1471-2105-9-471 (PMC2613932; doi:10.1186/1471-2105-9-471)
Supplement: Additional file 3 — Constrains used for MFOLD program for H/ACA-like molecules predicted by Psiscan. Examples of user-defined constraints for MFOLD program for H/ACA-like molecules. Our constraints prevent base pairing of the sequences which recognize the rRNA in the pseudouridylation pocket and force two nucleotides from stem II immediately after the pseudouridylation pocket to be base paired. [file 1471-2105-9-471-S3.doc]

**Constrains used for MFOLD program for H/ACA-like molecules predicted by Psiscan.**

Examples of user-defined constraints for MFOLD program for H/ACA-like molecules. Our constraints prevent base pairing of the sequences which recognize the rRNA in the pseudouridylation pocket and force two nucleotides from stem II immediately after the pseudouridylation pocket to be base paired.

TB3C2H1

p 11 0 6

p 52 0 5

f 17 51 1

TB8C4H2

p 12 0 5

p 53 0 6

f 17 52 1

TB9C6H1

p 12 0 6

p 56 0 6

f 18 55 1

TB10C5H3

p 11 0 6

p 51 0 5

f 17 50 1

TB11C5H3

p 16 0 3

p 51 0 5

f 19 50 1
