# Supplementary material for: Psiscan: a computational approach to identify H/ACA-like and AGA-like non-coding RNA in trypanosomatid genomes
Source: BMC Bioinformatics. 2008 Nov 5;9:471. doi: 10.1186/1471-2105-9-471 (PMC2613932; doi:10.1186/1471-2105-9-471)
Supplement: Additional file 4 — List of the sequences of intermediate results. Intermediate results consist of 25 sequences that were checked for H/ACA-like expression by primer extension analysis. [file 1471-2105-9-471-S4.doc]

**List of the sequences of intermediate results.**

**Intermediate results consist of 25 sequences that were checked for H/ACA-like expression by primer extension analysis.**

>TB10C5H3 (see figure 4 – sequence 206)

GAACGGTAACTCTCTGGAAACTCATCCCCTCTTTTCTGTAGATGTGTTTCCTCATCGGTTCCCGGAGAAAC

>171

TCATTCCACTTTCCAAAACCCTCACGTACGTGCGTTTTACTTCTGATGGAGGAGAAAA

>57

GTATTTATATCTTTTTCCCTCTGCATATATGACAATGAAGAGAAGTCAGGGTGAGGTGGGTAAGGGAGAGCT

>142

GAATTTGTTTCTTATTTCTCTTTTTCATTTAGTCAGTGGGCGGGGAACAAAACATAATAAACAAGACGA

>323

TAAGCGTTTTCTCCACCAAGTGTTTGTGCAACTGAACACATGCTGTTATTTAAAAGTAAAGAAAA

>246

CATTCCTTCGTTTGCTTTCACTCAAGCACTGATGGCTGTGGGAGCGAGGGGGATTAAGAACA

>265

CGACTTTGTTGTACATGCACTTGGATATCCACGTGAATATGTATGTTCGTAATGGAGACAAGCAGAGGAGAATC

>354

TTCCTTTTCCTTGCTTCTACAAAGTCGTGCTTGACGGTGGCATCCATAAAAGAAAAGAGGT

>651

TTCTTTGCGTGTTTCTTATATGAAATATGATAGGGGTGTTGCGCCTGCCTGGCTCTTCACCCAAGCAAATAAGAGGT

>1

CCAGTTTCAGAGAGACATACGACAGGATGAGTTGTGTGCTATTCTTTGTGATGGAGGAAACACAGATTT

>TB11C5H3 (see figure 4 sequence 16)

TAAGGTCAGTTCGAAGGCCCAGTACTTGTACCATTGTAAGTGCAATGTGGTTCAAAAGCTGACCAAGATTT

>3 ATATTGGTGGGGGAGGGGAGATGCAATGAGATTGTTATTCCACTTTCAAACTTGGTGCACAACCCTCGCAAGAAAA

>4

TCACCAATGCCAGCACCCGCCTCTGATGTTGTGGCTTGCGCCATCTGCTTGGAGCAATGGTCAGATCC

>5

ACAGAGGAGGAGAAGTGGCTGCGGCACCACCCCATGCTGTGCGGTGACGCCGTTTCATTCAGAATT

>6

GGAGTGCGTAGAGCTCGAGGGAGTGTCCCCGTGTTGGTGGTCCCGTTCCTCTTCAAGCGTAAGAAGC

>7 (see figure 4 sequence 7)

GCAGATGCCGGGAGCAGGGGAGTCATCCACAGGCAGACGGCTCTCGGCGTGCATTTTAGAGAGAA

>8

CCACTCTCACCCGAAGAGGACTCCTTCTACCGACAGGCAGTTGACCACTTCACACACTGCGAGGCCAGAGAG

>9

TGAGGAAGCTGAAGTTGTGCGTGAGTTGTGTGAAGCATCCGTGCGCGCGTGCGCTTCCTGAGACCT

>10

AAACGTCTTGGTCCATTTGAGCTTCATTGGGTTACGCTTCATACCAAAGTTCTTTCGGCACTTGGAGGCGCAGAAAC

>11

ACACTTATCAAAGACGTGCCACACCTTCACAGCAGGGTTGTGTTCGGATGCGCGATAAACAGAAAA

>12

GAATGCGTGGGAAGTTTTGTCGTTGGCGTCGGCAACTGTAGATAAACAGAGCAGAAGCGCAACCAGACGT

>13

CAACACCAGCATCAGCACCAGTGACAATTGTAACAATGCCACCACTGGGAGCAGTGGTGGCGGAGAGGA >14

TCACGCCATCCGTCGCCTCAAGGCTGATCCGTGCGCTGAAGGAGCCACCGCGTGATCGCAAGAAGGTGAAGAATA

>15

CAAGTGAAGCTGCTAGTGGCTTATGGCGCTCAGAGGACATGACACTGCTGCGGTTGACGATGCAGCGTGAGACGG

>TB3C2H1 (see figure 4 sequence 25)

TAATGAGGGTCTAAGAGCTGGGGACCGGAACCTTTCCATGTTCTTCCCAGTGTCATTGCCCTCAGAGA
