# Supplementary material for: Psiscan: a computational approach to identify H/ACA-like and AGA-like non-coding RNA in trypanosomatid genomes
Source: BMC Bioinformatics. 2008 Nov 5;9:471. doi: 10.1186/1471-2105-9-471 (PMC2613932; doi:10.1186/1471-2105-9-471)
Supplement: Additional file 5 — List of the sequences of final results. Final results consist of 19 sequences that were checked for H/ACA-like expression by primer extension analysis. [file 1471-2105-9-471-S5.doc]

**List of the sequences of final results.**

**Final results consist of 19 sequences that were checked for H/ACA-like expression by primer extension analysis.**

**List of the final results that were shown to be expressed (H/ACA-like and AGA-like)**

>TB8C4H2 (937)

AAACGAGGGACATCGTCGGGGGCTCTGATTCAACTTCTGTGGGGCCAAACCGAAACAACACCTTCGAAGATAT

>122

CTCTCTTTTTCCACTCGTTTTCCTCTTTCCGGGGAGAAGGAGGAGAAAGAGAGGAGGAAGGGAAGGAGAGAA

>109

GAACTCACTCTTGCCCACACCCTTGGAAACCGAAGAAGCAACGGGGGAGGGTGGAGAAGGTGGGGAAGACGT

>400

CTCTTTGTTTGCATCTCCACACTGGCCTCCGCGCACGTGGGTGGGTGTTTGTGTGCGTGGGTGCGAATGAGAGTC

>473

CAACTGCTCCATACTCCCCAGCGGCCACGCAACTGGCACGCCGGAAGGGGCATTTGGAGCGGCAGAGGA

>TB9C6H1(230)

CCCTCCGTTGTTTGTGCTTTTTGATATACTTGCTAATGCTTTGAAGTGTGTGAGGGGAAGGGGAGGAGGGAGAGAAAA

>299

GGTGAGGCGTTGACGCCGCGACCGCCTTAGCGAGACCTCAACGGCGCACGGGCGGTGCGTGGGGCGTCTTCAGAGGC

**List of the final results that were checked by primer extension, but were not found to be expressed**

>59

AAACTCCTCAACTACGTCCTTTCAGGTCTCGCCAAGACCTACACGAAGGGACAAAAAGGGGAAGAAGAACA

>164

TTTCCCGCTGACAACGTGGGTGGTACACCCTGGAGTAGCGGTAGCCGTCTCATCGCGGCACGTGGGTGCAGAAAT

>623

ACAGCGTGCGAGAACTGGAGACAGATGGCGTTGTCCCAGCCACCCACTCTACAGATGCCACGCATAGACTA

>829

TTCCCCTCTTCAGTTCCCTTCTCCCTCTCCTATCCTACGCTCCTTCCGTGGTAGTAGGGGCCAGGGAAGAGGGCGAGAAAA

>269

CTCGTTTCCTTTTAAACGCCTATAAGCAGCAAACTCATAAGGTTTATGCGGCGAAGGAAAGGGAAACAAAGAATA

>12

TCACGAATGGGTAGTCAGCTGCAGTCCGGCTTCACAAGTCATAGGCCACCGTGGCGTTACCCCATTCGAAGAGCC

>821 ACTTTCACATATTTTCGTATAATCGTGCATGTGCGTGTGCGTGTGCGTGTGAGAGTGAGAGTGAGAGTA

>299

GGTGAGGCGTTGACGCCGCGACCGCCTTAGCGAGACCTCAACGGCGCACGGGCGGTGCGTGGGGCGTCTTCAGAGGC

>607

TTCCTCCTTTCTTTCTCCTTGCAAAGGAGACATCCCTGCACTACTGAGCGAGACAGGAGGAGAAGAAAA

>1014

AACCCCTCTCACTCTTAGTGCGCCTGAGCCGGGGACTTTGAAGTGGCTTCAGACGGCGAGGAAAGGGGGATAGACTT

>622

AAACACCCACAGGTGTCTGACACTTAGGCATAACAGTGTAAAAAGGTGGGGATTAGTTACGGGTGGAAGAAGA
