# Supplementary material for: Psiscan: a computational approach to identify H/ACA-like and AGA-like non-coding RNA in trypanosomatid genomes
Source: BMC Bioinformatics. 2008 Nov 5;9:471. doi: 10.1186/1471-2105-9-471 (PMC2613932; doi:10.1186/1471-2105-9-471)
Supplement: Additional file 6 — List of the novel predicted H/ACA-like and C/D snoRNAs. The list consists of the novel H/ACA-like snoRNAs predicted by Psiscan method and of novel H/ACA-like and C/D snoRNAs predicted by manual exploration of clusters in T. brucei and L. major. [file 1471-2105-9-471-S6.doc]

**List of the novel predicted H/ACA-like and C/D snoRNAs.**

The list consists of the novel H/ACA-like snoRNAs predicted by Psiscan method and of novel H/ACA-like and C/D snoRNAs predicted by manual exploration of clusters in *T.brucei* and *L.major*.

>TB3C2H1

ATGTTGACAAATCGCGACAGCGGGAGCACAGTTTTCCCTTCCGAGGCAAAGCGCTAGATCCACCAACTACCCGTGGTATAATGAGGGTCTAAGAGCTGGGGACCGGAACCTTTCCATGTTCTTCCCAGTGTCATTGCCCTCAGAGA

>TB3C2H2

GCAGCAGAGATGTTTGAGGGGCTTTGCTGCCACGAAGTGTGGCCACAATAGCAAATACTGCGCCATAGGTACTCTGCAAGAAAT

>TB8C4C1

TGATGAGAGAAAGAGGGAAGGGAATGACCATCGGATTAATGAAAACATTTCATAGCACATGTGGAGCGGTGTAAATCCTTCTGTTTCTGA

>TB8C4H1

CATCTGTGGGCCTCACCCGGGTGATTAATGCACTTTAAAAGTGGGGAACGTGACACCCAAAGGGAGAAGG

>TB8C4H2 AAACGAGGGACATCGTCGGGGGCTCTGATTCAACTTCTGTGGGGCCAAACCGAAACAACACCTTCGAAGATAT

>TB9C6H1

TTATTTTTAGTATCAGTGTTTCATTTTTTTTTCTTTTTTCGTTTATCATCACTTTATTATTATTATTATTACGTCCCTCCGTTGTTTGTGCTTTTTGATATACTTGCTAATGCTTTGAAGTGTGTGAGGGGAAGGGGAGGAGGGAGAGAAAA

>TB9C6H2

TGTTTCTTTTTATTATTATTATTTGCTTACGTTCATAAAAAGTAGAGGAATGGAATGGAAAGAGATGG

>TB10C5H1

CTTTTTCTTTTATCTCGTTTTGTAGATCTCTACCACATGTGCGTATGCCAAAGGTGTAATTAAAGCAGGTAGGAGGAACGAGAGGA

>TB10C5H2

GGAGCAGACTACGTCTGTAATATGCGGAACCAACCTTCTCTGCACTTTACTGTAAATGTCTGCGAGATCA

>TB10C5C1

ACTTATGATGACAATATTATTTCTTACTTTCTGATCCGTGATTTGATTGACACAAGCACTGTCTGTGCGAACGTATCGAGAACCTGATT

>TB10C5H3

GAACGGTAACTCTCTGGAAACTCATCCCCTCTTTTCTGTAGATGTGTTTCCTCATCGGTTCCCGGAGAAAC

>TB11C5C1

TGTTGAGTGTGTGCGTGCGTGGGTGATGTCTTTTGTGGAACTCCAACCTGTACCTGCCGGGTCGCGGAACGAGATGCCGCGGAGCTGA

>TB11C5H1

CTCAACACCCACCAACCAGCCCCTTTCTGTGCGGTAGCTTGCACAATGGTGGCCAAAGTGTGGTGTGAGATTT

>TB11C5H2

CAAGCAGCATCAGTACAGCGGTGGTATCGCTAAGGCACCATGATACTTTCCGCTGGCGATGTGGCGAGATAC

>TB11C5C2

TGCTGTTTTCGTCTTCCTTGCGTTTTTTCCCCTCTATTTCGTGTTCCTTTCGCCTACAAAGTTCCCGGCGGTGACCCTGA

>TB11C5H3

TAAGGTCAGTTCGAAGGCCCAGTACTTGTACCATTGTAAGTGCAATGTGGTTCAAAAGCTGACCAAGATTT

>LM30C2H1

CCCGCACAGTTCGAAGGCCGTCATCCCCTGTACTACATCTTAGGGTGCTTTCGGTTCAAAGGCTGTGCCAGATAGAT

>LM36C4C1_a

TGATGATACCCTACTTGTTCTATTCAACAGAACACACGCACCAGCACACGCCCTCGCACACGCACTCCTTTGTGTGCGTGCGCCGGTGTGTGCTGGCGTGGGTGCATGATCGAACAGCCTCGTAGCAATACGTATCGAGAATCTGA

>LM36C4C1_b

TGATGATACTCTCTACTTGCTCATTTTTTTGCGAACAGATGTGCATGGCATCCCCACCTCTTCTGTGGATCCGCGCAGAACGCAAGATTGGCGGAAGCACTGCTTCACTGATGAGCTCCATAGCAATACGTATCGAGAACCTGA

>LM36C4H1_a

ACTACAACTCTCTGGATTCCTCGAGTCATTTAATTCTAGTGCAATCCTCATCGGTTGTCGAAGAGAT

>LM36Cs4H1_b

ACTACAACTCTCTGGATCCCTTGGGTCATTTCTCATTCCCAGTGCGATCCTCATCAGTTGTCGAAGAAGG

>Lm36C4C2

TGATGAGAGGCACCCCACGAGGGCGTCAGGGCCCTGCACATTCTCTCTGTGTCTGTGTGCGTGTGTGCGTGTGTGTGGGAGGGGGAAGGGAGCCACGCAGCCCCTCCCCCGTTCCCCTATCCCTCTGCCAAATGCCGAGCCACGTCTGA
