# Supplementary material for: Psiscan: a computational approach to identify H/ACA-like and AGA-like non-coding RNA in trypanosomatid genomes
Source: BMC Bioinformatics. 2008 Nov 5;9:471. doi: 10.1186/1471-2105-9-471 (PMC2613932; doi:10.1186/1471-2105-9-471)
Supplement: Additional file 7 — List of the random sequences that were analyzed by primer extension. The list consists of 15 sequences that were randomly selected from intergenic regions of the T. brucei genome. [file 1471-2105-9-471-S7.doc]

**List of the random sequences that were analyzed by primer extension.**

The list consists of 15 sequences that were randomly selected from intergenic regions of the *T.brucei* genome.

>1a (found to be expressed)

CTATGATAATAATAACTACACAAATATAGAAAAGTGAAAGAAAATGTGTGTAAATGAGAGGTACCGCTTCAACCAAATG

>2 AAGGGCAGAAGTGAAGAAAAGACAAATGGGAGGTAAAACGAAGTGAAGAGATTTGTAATAAACGCAAATTA

>3 AGACGGCAGAACACGGTGGCACGTTGAGCCAGACACTATTACCACGAGTCGATGAGAGCCGACAGAATCAACAAACAAACG

>4 CAGCGTTACGACTTTTCGTTAATTCCTTTTCTCTTCATTTATTTCCATATTTATTTCTACGCACAACATACATAAATGCAATACGATGCCCTT

>5

CATACTGTTGAAAAGAAGTGAGTGGAAGAAGGAAAGCGCATGCGTGTGAAGGAAGAAACATACAAAAAA

>1b

AAAGAAAATGATTGAATCAGCGAATTCGCCCTTGACTGAGTGTTTGTAAGTTCTCCAAGAGAG

>13

ATAGTACCTAGTAGGTTTCGTAACACCAGTAGAGGCGGAATGTATTTAGTTTTCTGCACAGATCT

>31

CTCACGTTACTCTCGTTTCAGCTTAACTAGAATAATTCAATTTGGCTCAAAGTGTAGAAGAGCT

>36

GACAATAGGGACAACAAAAGTAGGAGGGAGAACGTCATAGTTTGAGGAGTTGTGTTCAAAGAAGT

>58

TGATCTACCTTGAGTGAGTCGGCACGCACACTGCCAGCTGTGAGTTCTCGGTTGGAAAAAGATCC

>72

AAAGTGGAAAACGGTGCATGTTATGCTCTGGAACCCCACTACTTCGGACGTCTACGAAGAGAC

>105

CACGGATTTAGTTCAAAGGCCGCTAGTTATTAGGGAGAAGGTAAAATGTAGCGTTAGAGAAGA

>111 (found to be expressed)

CCAGCATGTTTTGCTCTTTGAGCTCAGTGGCATCGCCGGAGGAAATAGAAAGAGACGTGAGATGA

>158

TACGTATGGCCGATTTGCCACCACACAGTTTTGACAACGGGAGTATTAACCGTGTGCCGCAGAAGG

>167

TCTTGAGTTGCTAGTATGAAACCGTGTGTATTAATCCACGGTGAGTTACTGCTTGCAGACCA
